# Mitochondrial and lysosomal dysfunctions might be involved in the pathogenesis of the *CACNA1A-*related neurodevelopmental disorders according to in vitro studies

**DOI:** 10.1186/s40659-025-00655-w

**Published:** 2025-12-27

**Authors:** Miriam Kessi, Langui Pan, Baiyu Chen, Li Yang, Lifen Yang, Olumuyiwa A. Bamgbade, Guoli Wang, Jing Peng, Fei Yin, Fang He

**Affiliations:** 1 Department of Pediatrics, Xiangya Hospital, Central South University, Changsha, 410008 Hunan China; 2 Hunan Intellectual and Developmental Disabilities Research Center, Pediatrics, Changsha, China; 3 Clinical Research Center for Children Neurodevelopmental Disabilities of Hunan Province, Xiangya Hospital, Central South University, Changsha, China; 4 Department of Anesthesiology and Pharmacology, University of British Columbia, Vancouver, BC Canada

**Keywords:** *CACNA1A*, Neurodevelopmental disorders, Molecular mechanisms, Mitochondrial dysfunction, Mitochondrial fusion, Mitochondrial fission, Mitophagy, Lysosomal dysfunction

## Abstract

**Background:**

*CACNA1A* variants are associated with severe neurodevelopmental disorders (NDDs), but the underlying mechanisms remain unclear. Our goal was to investigate the molecular mechanisms through which these variants lead to intellectual disability (ID), autism spectrum disorder (ASD), epilepsy, and ataxia.

**Methods:**

Clinical information was collected from six pediatric patients. Molecular experiments were performed on transfected human embryonic kidney and Chinese hamster ovary cells to study the effect of these variants on mitochondrial and lysosomal function. RT-qPCR, Western blot, apoptosis assay, mitochondrial and lysosomal tracker fluorescence intensity, and mitochondrial calcium concentration tests were performed. Additionally, we examined the levels of reactive oxygen species (ROS), adenosine triphosphate (ATP), and mitochondrial enzymes and copy numbers.

**Results:**

We identified six variants that downregulated *CACNA1A* mRNA: p.D1644N, p.Y62C, p.G701R, p.R279C, p.R1664Q, and p.L1422Sfs*8. Five variants down-regulated Ca_v_2.1 protein expression, whereas, the p.R279C variant up-regulated it. All variants led to dysfunctions in the autophagy-lysosomal system: p.D1644N, p.R279C, and p.G701R variants blocked the fusion of autophagosomes and lysosomes while p.Y62C, p.R1664Q, and p.L1422Sfs*8 variants displayed increased lysosomal expression. The p.Y62C, p.G701R, p.R279C, p.R1664Q, and p.L1422Sfs*8 variants exhibited defective autophagy. The p.Y62C and p.D1644N variants disrupted mitochondrial function by downregulating mitochondrial enzyme activities and ATP levels, as well as by upregulating mitochondrial copy numbers, calcium levels, and ROS levels. Furthermore, the p.Y62C variant increased mitochondrial expression, fusion, and fission. In contrast, the p.D1644N variant decreased mitochondrial expression, fusion, fission, and mitophagy. The p.G701R, p.R279C, and p.R1664Q variants also interrupted mitochondrial function. These variants down-regulated mitochondrial enzyme activities, fusion and fission, the mitophagy process, and ATP levels while up-regulating mitochondrial copy numbers and ROS levels. The p.L1422Sfs*8 variant increased the expression, fusion and fission of mitochondrial proteins, while decreasing mitochondrial calcium levels and the mitophagy process. The p.R279C variant increased mitochondrial expression and calcium levels while enhancing apoptosis. The p.G701R variant decreased mitochondrial expression and calcium levels while enhancing apoptosis. The p.R1664Q variant increased mitochondrial calcium levels and enhanced apoptosis without changing mitochondrial expression.

**Conclusions:**

*CACNA1A* variants may alter mitochondrial and lysosomal function, resulting in the development of NDDs.

**Graphical abstract:**

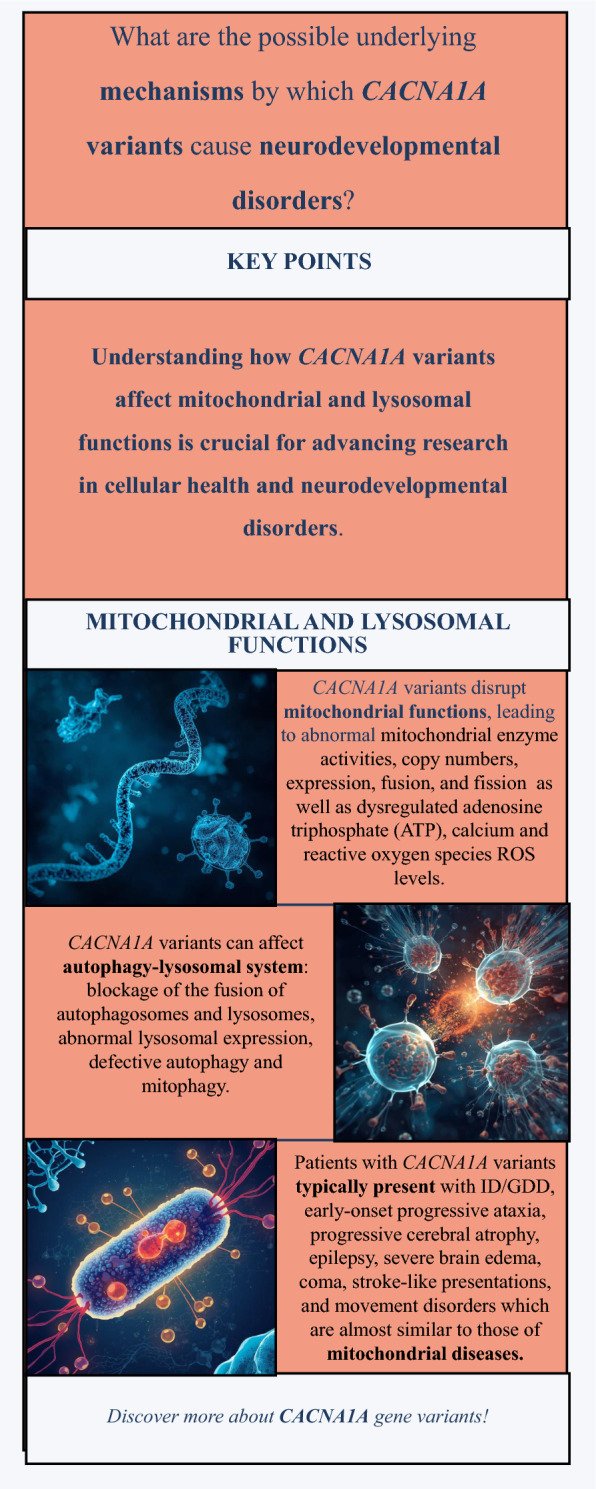

**Supplementary Information:**

The online version contains supplementary material available at 10.1186/s40659-025-00655-w.

## Introduction

*CACNA1A* (calcium voltage-gated channel subunit alpha1 A) encodes an alpha-1 subunit of a calcium channel (Ca_v_2.1 or P/Q-type) [1]. Ca_v_2.1 channels are widely distributed throughout the central nervous system (CNS), with high expression levels in the cerebral cortex, hippocampus, and the cerebellum [2]. *CACNA1A* variants are associated with a wide range of neurodevelopmental disorders (NDDs) including intellectual disability (ID)/global developmental delay (GDD), epilepsy, autism spectrum disorder (ASD), attention deficit hyperactivity disorder (ADHD), migraine, progressive ataxia, progressive cerebellar or cerebral atrophy, episodic ataxia, atypical Rett syndrome, and epileptic encephalopathy [3–5]. Some individuals with *CACNA1A* pathogenic variants may exhibit severe symptoms, including fatal refractory brain edema, stroke-like symptoms, recurrent loss of consciousness, coma, and visual impairment [6–8]. Furthermore, patients with severe phenotypes may die at a young age [9–12]. Currently, there is no specific treatment for this type of channelopathy. Calcium channel blockers are only partially effective in treating severe hemiplegic migraines caused by gain-of-function (GOF) variants. However, these medications are ineffective in treating NDDs and neuropsychiatric disorders [5, 13]. A previous study showed that *Cacna1a*-deficient mice exhibited rapidly progressive neurological deficits, including ataxia and dystonia, before dying four weeks after birth [14]. Interestingly, synaptic transmission continued in their hippocampal slices despite the absence of Ca_v_2.1 channels because N- and R-types calcium channels compensated for the influx of calcium ions [14]. Of concern, both GOF and loss-of-function (LOF) variants exhibit similar phenotypes in patients [3–5]. Consequently, it appears that an unknown, complex mechanism plays a role in the pathogenesis of NDDs, and conducting electrophysiological studies alone is insufficient.

Ca_v_2.1 channels are not only localized in the neuronal plasma membrane, but also in the cytosol. Their cytoplasmic aggregations can cause neurodegeneration [15]. Additionally, it was recently revealed that Cav2.1 channels are present on lysosomes, where they regulate fusion with autophagosomes and endosomes. This process is crucial for neuronal homeostasis [16]. Noteworthy, the function and regulation of Ca_v_2.1 channels found on lysosomes are independent of Ca_v_2.1 channels located on the plasma membrane [16]. Moreover, studies on *Cacna1a* mice with a spontaneous mutation have shown that cytoplasmic and lysosomal calcium levels are different [17]. Calcium ions can be stored in the mitochondria, endoplasmic reticulum and lysosomes [18, 19]. The endoplasmic reticulum is the most important of those stores [20]. The widespread distribution of Ca_v_2.1 channels and the presence of multiple calcium ion storage sites in neuronal cytosols, lysosomes, and mitochondria suggest that complex interactions of these channels may play an important role in *CACNA1A*-related NDDs pathogenesis. According to a few studies, mitochondrial dysfunctions have been detected in pediatric patients harboring *CACNA1A* variants. One patient with *CACNA1A* p.F1502del, a GOF variant, presented with congenital ataxia, epilepsy, GDD, abnormal eye movements, and severe hemiplegic migraines associated with hemispheric swelling [21]. Muscle biopsy tests revealed mitochondrial dysfunction in the form of partial deficits in complexes II, III, and IV of the enzyme system [21]. Another patient was diagnosed with refractory epilepsy and an early stroke. The patient was also a carrier of the *CACNA1A* p.L1692Q variant [22]. Muscle biopsy analysis showed mitochondrial depletion. Electron transport chain studies showed reduced respiratory chain complex activities [22]. Furthermore, patients with *CACNA1A* variants may present with a wide range of phenotypes resembling mitochondrial dysfunction, such as stroke, provoked seizures, early-onset progressive ataxia, migraine, epilepsy, GDD/ID, early-onset progressive cerebellar and/or cerebral dystrophy, and optic nerve atrophy [5]. Nevertheless, their underlying pathophysiological mechanisms remain unclear [5]. Previous studies have primarily focused on electrophysiological changes rather than molecular changes. Mitochondria and lysosomes depend on each other to maintain their proper structure and function [23], thus, they may be involved in the development of NDDs related to this gene.

The aim of our study was to explore the molecular mechanisms by which six *CACNA1A* variants cause NDDs. Specifically; we examined the roles of mitochondria and lysosomes in the pathogenesis of NDDs via several experiments performed on transfected human embryonic kidney (HEK 293) and Chinese hamster ovary (CHO) cells. Our novel findings may improve our understanding of the molecular mechanisms of these disorders and pave the way for identifying potential treatment targets.

## Materials and methods

### Ethical clearance and subjects

The study complies with all ethical regulations and was approved by the Institutional Ethics Committee of Xiangya Hospital, Central South University (approval number 202310892). Written informed consents were obtained from the parents or guardians of the patients. Patients diagnosed with *CACNA1A*-related NDDs at Xiangya Hospital, Central South University, from 2018 to 2023 were enrolled in the study. Two pediatric neurologists evaluated patients. The following comprehensive clinical data was collected: prenatal, perinatal, postnatal, family, and developmental histories, seizure types, neurodevelopmental symptoms, video electroencephalography (EEG) and neuroimaging results, developmental milestones, treatment regimens and outcomes. Genetic test results was collected and analyzed by a geneticist.

### Sequencing, analysis and interpretation

After receiving signed informed consent forms, blood samples were collected from the patients and their biological parents. According to the previous protocol [24], genomic deoxyribonucleic acid (DNA) was extracted. Whole exome sequencing was performed for all six cases in a trio setting. Sanger sequencing was used to validate the parental origin of the variants. Exome library preparation, sequencing, bioinformatics filtering, and data analysis were conducted. We carried out a comprehensive comparison of our variants with established variants by utilizing resources such as the Genome Aggregation Database (https://gnomad.broadinstitute.org), InterVar (http://wintervar.wglab.org/), ClinVar (https://www.ncbi.nlm.nih.gov/clinvar/), and the Human Gene Mutation Database (https://www.hgmd.cf.ac.uk/ac/index.php). We collected and interpreted the genetic results in accordance with the variant curation guidelines published by the American College of Medical Genetics in 2015 [25].

### Cell culture, plasmid construction and transfection

HEK 293 and CHO cells were purchased from the Shanghai Cell Bank of the American Type Culture Collection. Cells were cultured in Dulbecco’s Modified Eagle Medium (DMEM) basic and DMEM/F12 (Thermo Fisher Scientific), which was supplemented with 5% inactivated fetal bovine serum, 1% penicillin–streptomycin, and maintained at 37 °C in 5% CO₂. The pcDNA3.1-T2A-EGFP2 was constructed for the wild type (WT) and variants. The WT as well as the p.G701R, p.R279C, and p.D1644N variants were constructed by the YouBio Biological Technology Co., Ltd. in Changsha, China, while the p.Y62C, p.L1422Sfs*8, and p.R1664Q were constructed by the TsingKe Biological Technology. The β3 + α2/δ1 Ca_v_2.1 subunits were gifts from the Hunan Normal University. According to the manufacturer’s instructions, t plasmids were transfected into HEK 293 and CHO cells using Lipofectamine 2000 (Invitrogen; Thermo Fisher Scientific, Inc.). Specifically, we used CHO cells for the immunofluorescence (mitochondrial and lysosomal staining) instead of HEK 293 cells because CHO cells can produce much clearer images. Whereas, all other experiments were performed in the HEK 293 cells.

### RNA extraction and real‑time quantitative PCR (RT-qPCR)

Primers for the *CACNA1A* variants were constructed using Primer Bank (https://pga.mgh.harvard.edu/primerbank). Primers for *CACNA1A* and *ACTIN* are presented in the Supplementary Table 1. Ribonucleic acids (RNAs) were extracted using the E.Z.N.A. Total RNA Kit (Omega) at or after 48 h post-transfection as described before [26]. Approximately 1000 ng of RNA from each mutant was reverse-transcribed using the Hifair II 1st Strand cDNA Synthesis Super Mix for Quantitative PCR (qPCR) kit according to the manufacturer’s instructions. RT-qPCR was performed using SYBR Green (Yeasen, China) on an ABI 7500 (Applied Biosystems, Thermo Fisher Scientific, Inc.). The relative mRNA expression of the target genes was calculated using the 2^ (− ΔΔ) CT method.

### Protein extraction and Western blot

Whole cell protein was extracted at or after 48 h post-transfection as described before [26] using a mixture of Radio Immunoprecipitation Assay (RIPA) lysis buffer and henylmethanesulfonyl fluoride (PMSF). The protein concentration was measured using a bicinchoninic acid (BCA) protein assay kit (Pierce). Equal amounts of 35 μg of protein from each sample were loaded onto a 12.5% sodium dodecyl sulfate polyacrylamide gel and separated by electrophoresis (80 V for 1 h, then 120 V for 1–2 h). Proteins were then transferred to 0.45 µm polyvinylidene difluoride membranes (Millipore) at 300 mA for 105 min. Membranes were blocked with protein-free rapid blocking buffer (product code 20B10) at room temperature for 30 min. Membranes were then incubated with primary antibodies at 4 °C overnight in a shaker machine to detect the effects of different variants on autophagy proteins (LAMP1, LC3II, Beclin-1, and p62), mitophagy proteins (PINK1 and PARKIN); mitochondrial fusion and fission proteins (OPA1 and DRP1); mitochondrial enzyme proteins (MT-CO1 and SDHA); and endoplasmic reticulum stress proteins (DDIT3/CHOP). After washing membranes three times for 10 min in phosphate-buffered saline mixed with Tween-20 (PBST) solution, depending on the origin of the primary antibodies, membranes were incubated with either HRP-conjugated AffiniPure goat anti-mouse secondary antibody or HRP-conjugated AffiniPure goat anti-rabbit secondary antibody at room temperature for one hour. Notably, western blots for the proteins of interest were cut from the same membrane for primary antibody incubation. Supplementary Table 2 provides detailed information on the antibodies used in this study. Chemiluminescence signals of target proteins were visualized using a New Super ECL Assay (cat. no. KGP1127-KGP1128) in a ChemiDoc XRS + system (Bio-Rad Laboratories, Inc.). The densitometric values of the blots were analyzed using Image J.

### Bafilomycin A1 treatment

HEK293 cells that had been transfected with WT and mutant plasmids were treated with the lysosome inhibitor Bafilomycin A1 at 100 nM for 12 h, within 36–48 h of transfection as previously described [27]. Dimethyl sulfoxide (DMSO; #5.43900, Sigma) was used as the control, and Bafilomycin A1 (cat. no. HY-100558, MedChemExpress) was used as the target. P62 and LC3-II/LC3-I autophagy markers were analyzed by Western blot.

### Intracellular reactive oxygen species level detection

The Ab113851 DCFDA/H2DCFDA cellular reactive oxygen species (ROS) assay kit was used to measure ROS levels in transfected HEK 293 cells at or after 48 h post-transfection [28]. ROS production was monitored using the ROS-sensitive fluorescent probe 5-amino-2,3-dihydro-1,4-phthalazinedione (3-aminophthalhydrazide, or luminol; Sigma) according to the manufacturer’s instructions. A microplate reader (Biotek Synergy H1, USA) was used for analysis.

### Determination of the ATP production rate

The adenosine triphosphate (ATP) production rate was determined using an ATP bioluminescence assay kit (CLS II). At or after 48 h post-transfection, cells were incubated at 100 °C for two minutes with a volume nine times that of the boiling 100 mM Tris and 4 mM ethylenediaminetetraacetic acid (EDTA) solution at pH 7.75 as described before [28]. Cells were then centrifuged at 1000 g for one minute, after which the supernatant was transferred to a new centrifuge tube and placed on ice. Then, 50 μL of the supernatant or ATP standard was transferred to a white 96-well plate. Next, 50 μL of luciferase working solution was added to each well, and the luminosity of each well was measured using a microplate reader (Biotek Synergy H1, USA). The luminosity of each well was then integrated for 10 s to calculate the average.

### Determination of mitochondrial complex I activity

The complex 1 activity was measured according to the instructions provided in the complex I enzyme activity assay kit (Colorimetric, Abcam, catalog number ab109721, available at http://www.abcam.com/ab109721). The transfected cells were washed twice with phosphate-buffered saline (PBS) over ice at or after 48 h post-transfection as described before [28], and then detergent was used to extract proteins. The detergent extracts of the prepared samples were loaded onto the plates and incubated for three hours at room temperature. Plate wells were washed with washing buffer three times. Then, 200 μL of the assay solution was added to each well. The optical density (OD₄₅₀) was measured in kinetic mode with a microplate reader (BioTek Synergy H1, USA) at room temperature for up to 30 min.

#### Mitochondrial copy numbers

The determination of mitochondrial DNA copy numbers was performed at or after 48 h post-transfection as described before [28]. After extracting genomic DNA using the E.Z.N.A. tissue DNA kit (Omega Bio-Tek), we determined the relative level of mitochondrial DNA copy numbers by RT-qPCR according to the SYBR Green fluorescent dye instructions (Yeasen, China). Target genes included mitochondrial (*mt-ND1*, *hMito-1*, *h-16S RNA-1*, and *mt3212*) and nuclear (*B2M*). Supplementary Table 3 lists primers used to check mitochondrial copy numbers. Triplicate amplifications of the mitochondrial and nuclear products were performed. We calculated mitochondrial/B2M copy number ratio for each sample and compared the relative copy number ratios between samples.

#### Mito-tracker red staining

CHO cells were washed twice with PBS at or after 48 h post-transfection as described previously [28]. Then, cells were stained with MitoTracker Red CMXRos (Invitrogen, Thermo Fisher Scientific). MitoTracker Red was added directly to the cell culture medium at a ratio of 1:10,000, after which cells were incubated for 30 min. Cells were washed twice with PBS, observed, and photographed using a Zeiss inverted fluorescence microscope with a 63 × oil objective. The photographs were analyzed using ImageJ.

#### Lyso-tracker red staining

Cells were stained with LysoTracker Red DND-99 (Lot Number 2204208, Invitrogen, Thermo Fisher Scientific) at or after 48 h post-transfection as described in another study [28]. LysoTracker Red was added directly to the cell culture medium at a ratio of 1:10,000 and incubated for 30 min. Cells were then washed twice with PBS, observed and photographed using an inverted fluorescence microscope (ZEISS Technology Co., Ltd.) with a 63 × oil objective. Photographs were analyzed using Image J.

#### Mitochondrial calcium concentration test

Mitochondrial calcium ion levels were determined using Rhod-2 AM. The transfected cells with WT and mutants were rinsed three times with Hank’s balanced salt solution (HBSS) and stained with a 5 μM solution of Rhod-2 AM diluted in HBSS at 37 °C for five minutes in the dark at or after 48 h post-transfection as described in previous study [28]. Finally, live cells were rinsed three times with HBSS and incubated at 37 °C for ten minutes in the dark. Cells were then analyzed using a flow cytometer (FACScan; BD Biosciences). Calcium ions levels of each variant were then compared to those of WT.

#### Statistical analysis

Data entry and processing were performed using GraphPad Prism 7.0 software. Outliers were checked for and removed if present. The Kolmogorov–Smirnov or Anderson–darling and D’Agostino-Pearson omnibus tests were used to test the normality of the data. The Mann–Whitney test was used to analyze data that did not pass normality test. A two-unpaired parametric t-test was used to analyze data that passed normality test. *P* < 0.05 indicated a statistically significant difference (*P < 0.05, **P < 0.01, ***P < 0.001, and ****P < 0.0001, as shown in the figures and figure legends).

## Results

### Clinical features and genetic results

Of the six cases, three were males. The age of disease onset ranged from one to ten years. Three patients presented with seizures, including two of focal seizures and one of absence seizures. Of the individuals with seizures, one presented with status epilepticus, and two had a history of febrile seizures. Overall, five children had ID/GDD, five ataxia, and two ASD. Of the three cases with seizures, two had ID/GDD prior to the onset of seizures. Two patients had profound ID/GDD, one severe and two mild. Three cases had cerebellar atrophy (Supplementary Table 4). The six patients carried six *CACNA1A* variants: four de novo and two inherited (p.G701R, p.R279C, p.D1644N, p.Y62C, p.L1422Sfs*8, and p.R1664Q; Supplementary Table 5). These variants can also be found in ClinVar (SCV003930352–SCV003930359).

The de novo p.Y62C variant (pathogenic) was found in patient 1, who presented with focal seizures, status epilepticus, severe GDD, ASD, and progressive ataxia. This variant has been reported in two previous cases who presented with focal epilepsy and status epilepticus [29, 30]. The novel p.L1422Sfs*8 variant, which was inherited from the mother, was found in patient 2, who presented with episodic ataxia type 2 (EA2). The whole exome sequencing revealed two *CACNA1A* variants: p.T2448I, which was inherited from unaffected father and was considered as a variant of unknown significance (VUS); and p.L1422Sfs*8, which was inherited from the mother with mild symptoms (paroxysmal dizziness and fatigue), and was considered as a pathogenic variant. Patient 2’s father and siblings had no symptoms (Supplementary Fig. 1). Some evidence in the literature suggests that EA2 is associated with LOF variants, such as deletion, in-frame insertion, and truncating mutations [5]. Patient 3 had p.R1664Q (pathogenic) variant and presented with progressive ataxia and mild ID. Six similar cases have been reported in the literature. The first case presented with mild ID and EA2 [31]. The second case presented with severe GDD, speech delay, hypotonia, behavioral issues, hyperreflexia, eye movement disorder, and constipation [32]. The third case presented with severe GDD, persistent ataxia, dysarthric speech, hypotonia, strabismus, myopia, and astigmatism [32]. The fourth and fifth patients presented with GDD, dysarthria, hypotonia, aggression, ocular apraxia, and hyperextensible joints [32]. The sixth case presented with progressive cerebellar ataxia, hypotonia, tremor, exotropia, and nystagmus [33].

Patient 4 who presented with absence seizures, mild ID, EA2, dizziness, loss of consciousness and memory, paroxysmal limb weakness, and gait instability was found to have the p.R279C variant, which was inherited from an unaffected father. This variant had been reported as an inherited variant in nine other individuals. The first patient presented with atypical absence seizures, severe GDD, hypotonia, tremor/myoclonus, and hypermetropia [34]. The second patient presented with ataxia, ASD, ADHD, depression, anxiety, GDD, astigmatism, nystagmus, and paroxysmal tonic upgaze [35]. The third patient presented with epilepsy (absence, myoclonic, and generalized seizures), ataxia, ID, feeding difficulties, and hypotonia [36]. Fourth, fifth and sixth patients presented with ID, EA2, dysarthria, and vertigo [36]. The seventh patient presented with epileptic seizures, ID, dysarthria, nystagmus, and speech delay [36]. The eighth patient presented with progressive ataxia, ID, and permanent gait disorders [36]. The last patient presented with focal epilepsy, ID, progressive ataxia, and permanent gait disorders [36]. The novel de novo p.G701R (pathogenic) variant was found in patient 5, who presented with profound ID, ASD, and progressive ataxia. The novel de novo p.D1644N variant was found in patient 6, who presented with focal seizures, profound ID, and progressive ataxia.

### *CACNA1A* variants reduced mRNA and protein expression

**Fig. 1:**
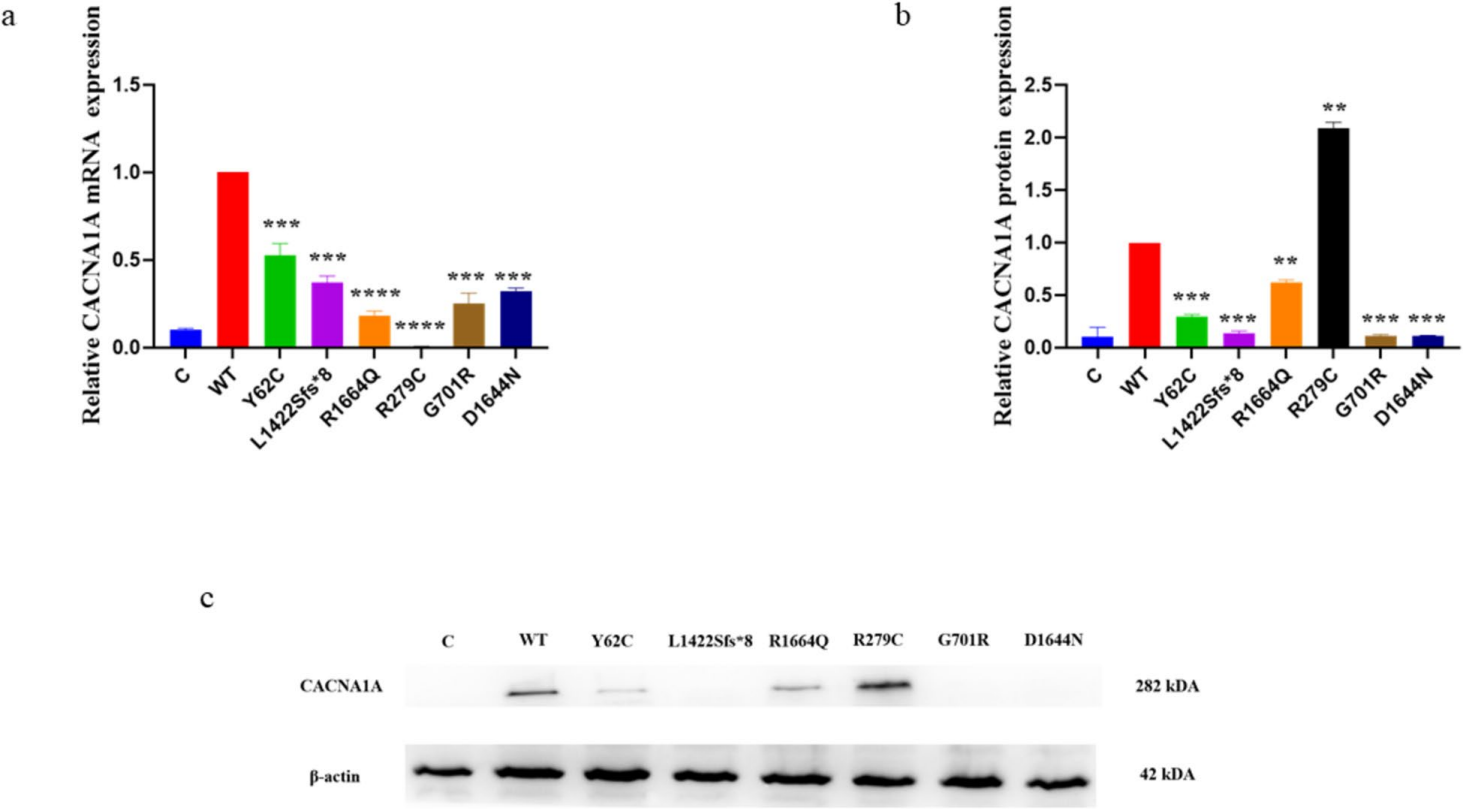
*CACNA1A* mRNA and total protein expressions in the HEK293 cells. A two-unpaired parametric t-test was used for the comparison between WT and each mutant. **a** Histogram showing relative *CACNA1A* mRNA expression. **b** Histogram showing the relative total Ca_v_2.1 protein values of respective immunoblots. **c** Immunoblots of the Ca_v_2.1 protein expression. **P < 0.01, *** *P* < 0.001, **** *P* < 0.0001, C, untransfected negative control; WT, wild type. Values represent mean ± SEM. Twelve replicates were run for the mRNA and eight replicates for the protein

The D’Agostino & Pearson test confirmed normal distribution of 12 replicates of mRNA data. Thereafter, WT was compared to each variant using a two-unpaired parametric t-test. Consequently, we found that all variants reduced *CACNA1A* mRNA expression compared to WT (Fig. 1a). Shapiro–Wilk test confirmed normal distribution of eight replicates of protein data. Thereafter, WT was compared to each variant using a unpaired parametric test. Thus, it was revealed that p.Y62C, p.L1422Sfs*8, p.R1664Q, p.G701R, and p.D1644N exhibited significantly lower protein expression than WT, while p.R279C exhibited higher expression than WT (Fig. 1a and b). With the exception of p.R279C, these results suggest that our variants might have had reduced transcription machinery. The p.R279C variant exhibited the lowest mRNA expression and the highest protein expression, suggesting the activation of auto-regulatory mechanisms [37].

### Variants affected autophagy-lysosomal system

**Fig. 2:**
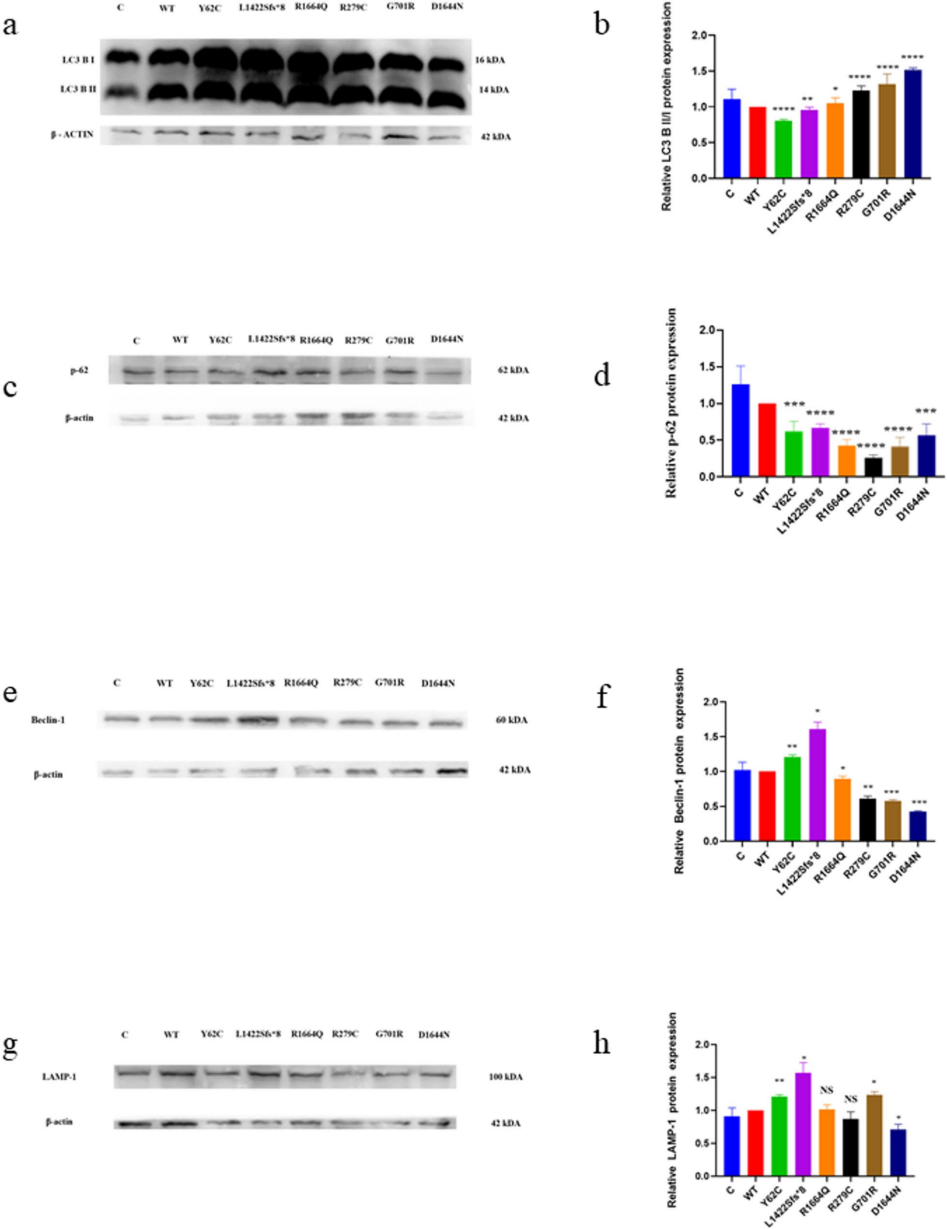
Western blot analysis results for the autophagy—lysosomal system in the HEK293 cells. A two-unpaired parametric t-test was used for the comparison between WT and each mutant. **a** Immunoblots of the LC3 II/I protein. **b** Histogram showing the relative LC3 B II/I protein values of respective immunoblots. **c** Immunoblots of the p62 protein. **d** Histogram showing the relative p62 protein values of respective immunoblots. **e** Immunoblots of the Beclin-1 protein. **f** Histogram showing the relative Beclin-1 protein values of respective immunoblots. **g** Immunoblots of the LAMP-1 protein. **h** Histogram showing the relative LAMP-1 protein values of respective immunoblots. Values represent mean ± SEM of five independent experiments. * *P* < 0.05, ** *P* < 0.01, *** *P* < 0.001, ****P < 0.0001, NS, non-significant; C, untransfected negative control; and WT, wild type

We examined whether our variants affected the autophagy-lysosomal system. Using RT-qPCR and Western blot analysis, we examined several autophagy-lysosomal pathway markers. This pathway is a key mechanism for degrading intracellular macromolecules. These catabolic products can be used by the cell to produce energy or other macromolecules [38]. This pathway begins with the formation of new double-membrane organelles (autophagophores) and ends with the fusion of autophagosomes and lysosomes to form autophagolysosomes, which degrade the contents of the autophagosomes. Cytosolic LC3-I is conjugated with phosphatidylethanolamine to form LC3-II [38]. This conjugate is then recruited to autophagosomal membranes, where it forms autophagosomes [39]. Up-regulation of the LC3-II lysosomal turnover marker signifies starvation-induced autophagic activity, while up-regulation of LC3-I implies nutrient richness [39]. The D’Agostino & Pearson and Kolmogorov–Smirnov tests confirmed normal distribution of 12 replicates of LC3-II/I protein expression data. Thereafter, WT was compared to each variant using a two-unpaired parametric t-test. Consequently, p.Y62C and p.L1422Sfs*8 exhibited significantly lower LC3-II/I protein expression than WT (Fig. 2a, b). Both p.Y62C and p.L1422Sfs*8 demonstrated defective autophagy [40]. On the other hand, p.R1664Q, p.R279C, p.G701R, and p.D1644N showed significantly higher levels of LC3-II/I protein expression than WT (Fig. 2a, b). An increase in LC3-II indicates autophagosome accumulation in the p.R1664Q, p.R279C, p.G701R, and p.D1644N variants [40]. Previous studies have shown that the amount of LC3-II correlates with the number of autophagosomes. An elevated amount of LC3-II indicates a buildup of autophagosomes but does not ensure autophagic degradation [40].

p62/SQSTM1 is a classical autophagy receptor that plays a role in both autophagosome formation and antioxidant stress [41]. p62 can bind directly to LC3, leading to protein degradation by autophagy. ROS can also induce this process. p62 levels increase when autophagy is inhibited and decrease when autophagy is induced [42]. p62 is connected to Parkin and is important for regulating mitophagy [42]. Therefore, abnormal p62 can affect the balance of mitophagy and disturb mitochondrial quality control [43]. The Shapiro–Wilk test confirmed normal distribution of five replicates of p62 protein expression data. Thereafter, WT was compared to each variant using a two-unpaired parametric t-test. As a result, p.Y62C, p.L1422Sfs*8, p.R1664Q, p.R279C, p.D1644N, and p.G701R expressed lower levels of p62 than WT (Fig. 2c, d).

Beclin-1 controls autophagy by forming complexes with various proteins [44]. Bcl-2 can interact with Beclin-1, which leads to the inhibition of Beclin-1-mediated autophagy in the endoplasmic reticulum [44]. Apoptotic caspases can cleave Beclin-1, resulting in its inactivation and reduced autophagy [45]. Beclin-1 modulates endocytic trafficking and LC3-associated phagocytosis [45]. Beclin-1 dysfunction can cause many conditions, including neurodegeneration and cancer [46]. The Shapiro–Wilk and Kolmogorov–Smirnov tests confirmed the normal distribution of five replicates of Beclin-1 protein expression data. Thereafter, WT was compared to each variant using a two-unpaired parametric t-test. Both p.Y62C and p.L1422Sfs*8 variants had significantly higher Beclin-1 protein expression than WT, whereas p.R1664Q, p.R279C, p.G701R, and p.D1644N showed significantly lower expression than WT (Fig. 2e, f).

**Fig. 3:**
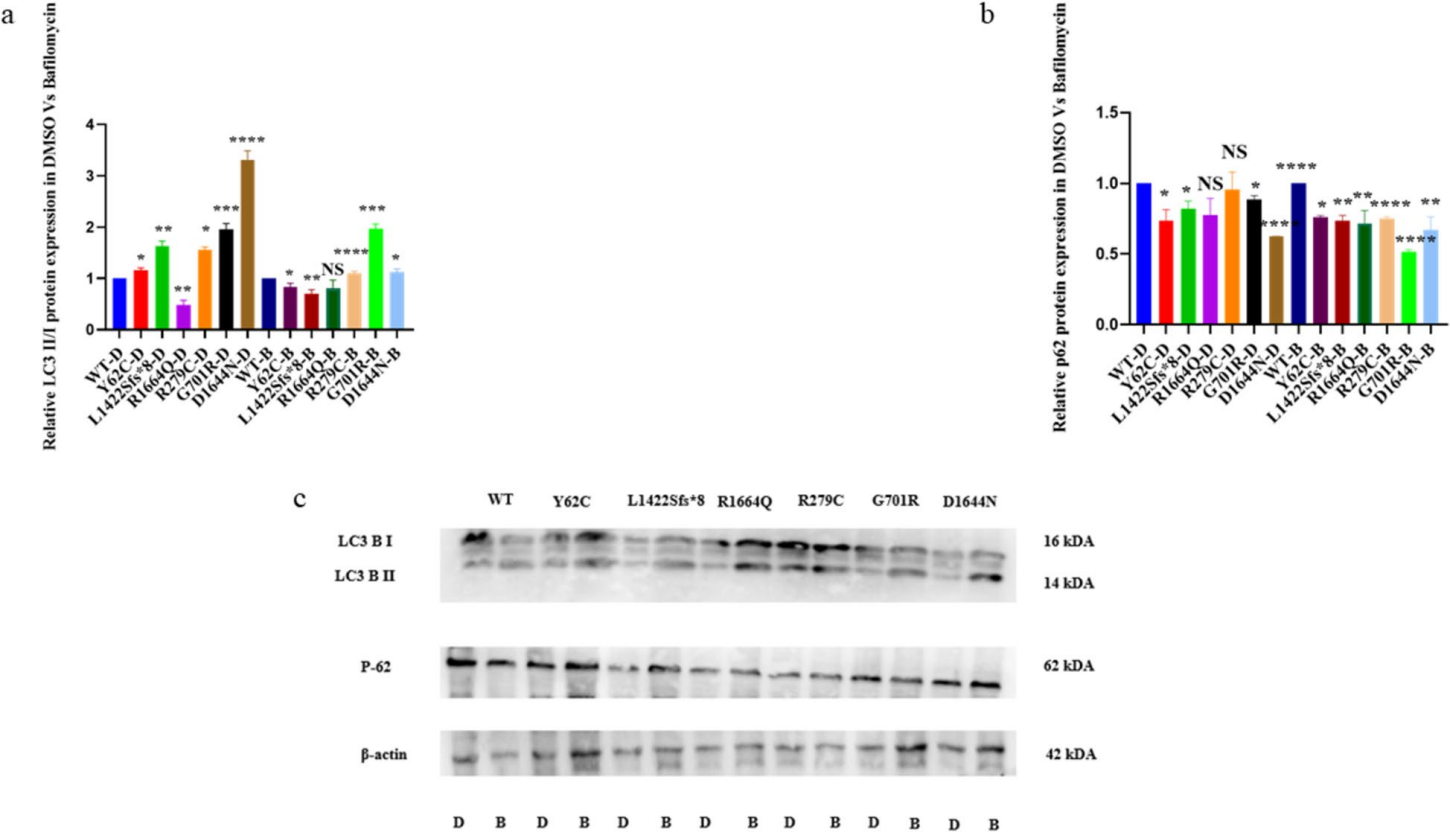
Western blot analysis of the LC3 B II/I and p.62 after treatment with Bafilomycin A1 in the HEK293 cells. WT with either DMSO or Bafilomycin A1 was compared to each variant with DMSO or Bafilomycin A1, respectively using Mann-Whitney test. a Histogram showing relative LC3 B II/I protein expression. b Histogram showing the relative p-62 protein expression. c Immublots of LC3 B II/I and p62 proteins. Values represent mean ± SEM of 5 independent experiments. * P < 0.05, ** P < 0.01, *** P < 0.001, ****P < 0.0001, B, Bafilomycin A 1; D, DMSO; NS, non-significant; and WT, wild type.

LAMP-1 is distributed in autophagic and endolysosomal organelles [47]. LAMP-1 is a biomarker of lysosomal biogenesis [48]. LAMP-1 lysosomal membranes fuse with LC3 autophagosomal membranes during the autophagy process [49]. The Shapiro–Wilk and Kolmogorov–Smirnov tests confirmed normal distribution of five replicates of LAMP-1 protein expression data. Thereafter, WT was compared to each variant using a two-unpaired parametric t-test. Levels of LAMP-1 protein were higher for p.Y62C, p.L1422Sfs*8, and p.G701R compared to WT, while p.D1644N showed significantly lower levels of expression (Fig. 2g, h). Neither p.R1664Q nor p.R279C showed significant changes compared to wild type (Fig. 2g, h). Bafilomycin A1 is an autophagy inhibitor. After removing outliers from five replicates of LC3 B II/I protein expression following treatment with DMSO and Bafilomycin A1, we found that the results were not normally distributed. Therefore, the WT variant was compared to each of the other variants with either DMSO or Bafilomycin A1 using the Mann–Whitney test. Since most variants showed activation of the autophagy-lysosomal pathway, we carried out a trial to treat transfected cells with Bafilomycin A1. However, p.G701R, p.R279C, and p.D1644N variants showed significantly higher levels of LC3 B II/I protein than WT after treatment with Bafilomycin A1. In contrast, p.Y62C and p.L1422Sfs*8 variants showed significantly lower LC3 B II/I protein expression than WT (Fig. 3a and c). The LC3-II/I protein levels for the p.R279C, p.G701R, and p.D1644N variants remained unchanged (higher than WT) after treatment with Bafilomycin A1, implying that autophagosome accumulation might have occurred due to the inhibition of autophagic degradation (blockage of autophagosome-lysosome fusion) [40]. The p.Y62C and p.L1422Sfs*8 variants remained unchanged (lower expression than WT) after treatment with Bafilomycin A1, which signifies defective autophagy [40]. The p.Y62C, p.L1422Sfs*8, p.R1664Q, p.R279C, p.G701R, and p.D1644N variants showed significantly lower p62 protein expression than WT (Fig. 3b and c).

After removing outliers from five replicates of p62 protein expression following treatment with DMSO and Bafilomycin A1, we found that the results were not normally distributed. Therefore, the WT variant was compared to each of the other variants with either DMSO or Bafilomycin A1 using the Mann–Whitney test. The p62 protein expression remained unchanged for all variants after treatment with Bafilomycin A1, which signifies defective autophagy [50].

**Fig. 4:**
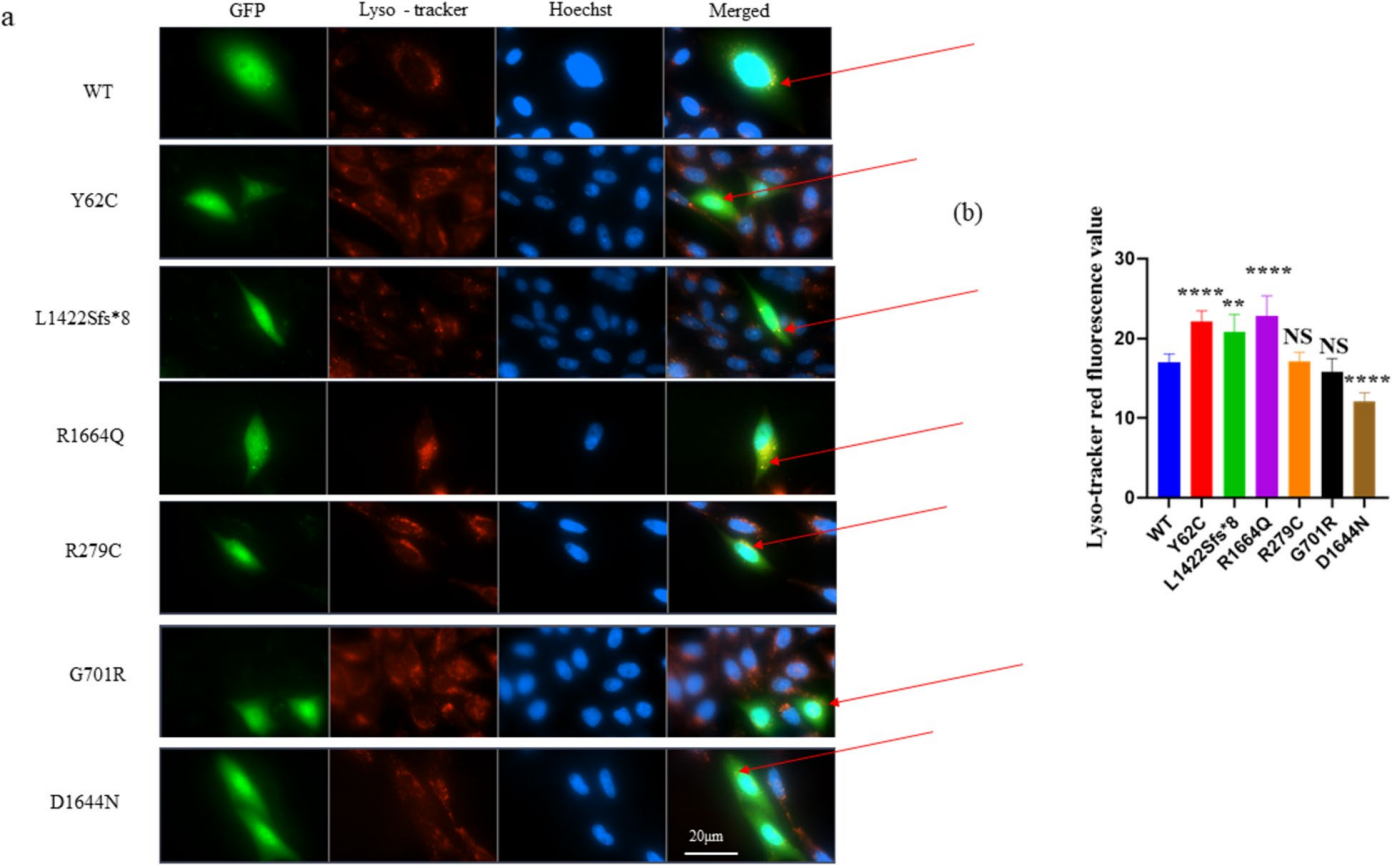
**a** Fluorescence microscopy for the lyso-tracker red staining in CHO cells. A two-unpaired parametric t-test was used for the comparison between WT and each mutant. **b** Histogram showing relative lyso-tracker red staining fluorescence. Besides, Ca_v_2.1 protein might co-localize with lysosomes as shown with the red arrows. Scale bar, 20 μm. NS, non-significant; WT, wild type. Data and images shown are representative of 4 independent experiments with at least 50 cells. ** *P* < 0.01, ****P < 0.0001, NS, non-significant; and WT, wild type

We used CHO cells to investigate the effects of the variants on lysosome expression. After removing outliers from a sample of > 50 cells per group, we found that the results were distributed normally according to Anderson–Darling test, Agostino & Pearson test, Shapiro–Wilk test or Kolmogorov–Smirnov test. Therefore, WT was compared to each variant using a two-unpaired parametric t-test. We found that Ca_v_2.1 protein might co-localize with lysosomes (Fig. 4a). The p.Y62C, p.L1422Sfs*8, p.R1664Q, and p.D1644N variants demonstrated statistically significantly lower lysosomal intensity than WT, while the p.R279C and p.G701R variants showed no significant change (Fig. 4b).

### Variants induced mitochondrial dysfunction

**Fig. 5:**
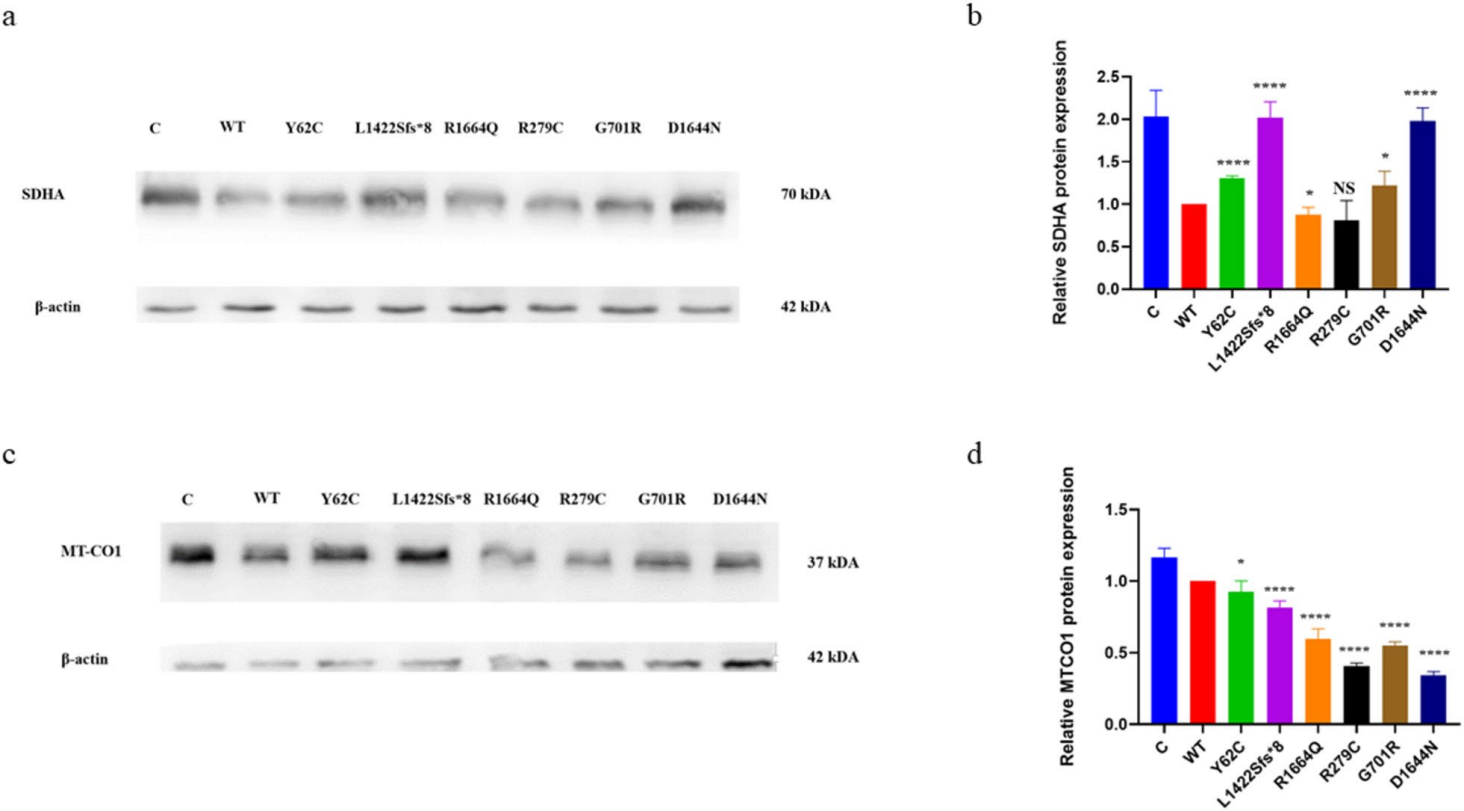
Mitochondrial dysfunction protein markers in the HEK293 cells. A two-unpaired parametric t-test was used for the comparison between WT and each mutant. **a** Immunoblots of the SDHA protein. **b** Histogram showing the relative SDHA protein values of respective immunoblots. **c** Immunoblots of the MT-CO1 protein. **d** Histogram showing the relative MT-CO1 protein values of respective immunoblots. Values represent mean ± SEM of 5 experiments. * *P* < 0.05, ****P < 0.0001, NS, non-significant; C, untransfected negative control; and WT, wild type

We examined several mitochondrial genes and proteins to determine if there was any dysfunction. The SDHA is a complex II mitochondrial respiratory chain protein. The Shapiro–Wilk test confirmed normal distribution of five replicates of SDHA protein expression data. Thereafter, WT was compared to each variant using a two-unpaired parametric t-test. Consequently, p.Y62C, p.L1422Sfs*8, p.G701R, and p.D1644N variants showed statistically significant higher SDHA protein expression than WT, while the p.R1664Q variant expressed lower protein that WT (Fig. 5a, b). The p.R279C variant showed no change compared to WT (Fig. 5a, b). MT-CO1 is the final enzyme in the mitochondrial electron transport chain that determines oxidative phosphorylation. The Shapiro–Wilk test confirmed normal distribution of five replicates of SDHA protein expression data. Thereafter, WT was compared to each variant using two-unpaired parametric t-test. Expression of the MT-CO1 protein was lower than the WT level for all variants (Fig. 5c, d).

**Fig. 6:**
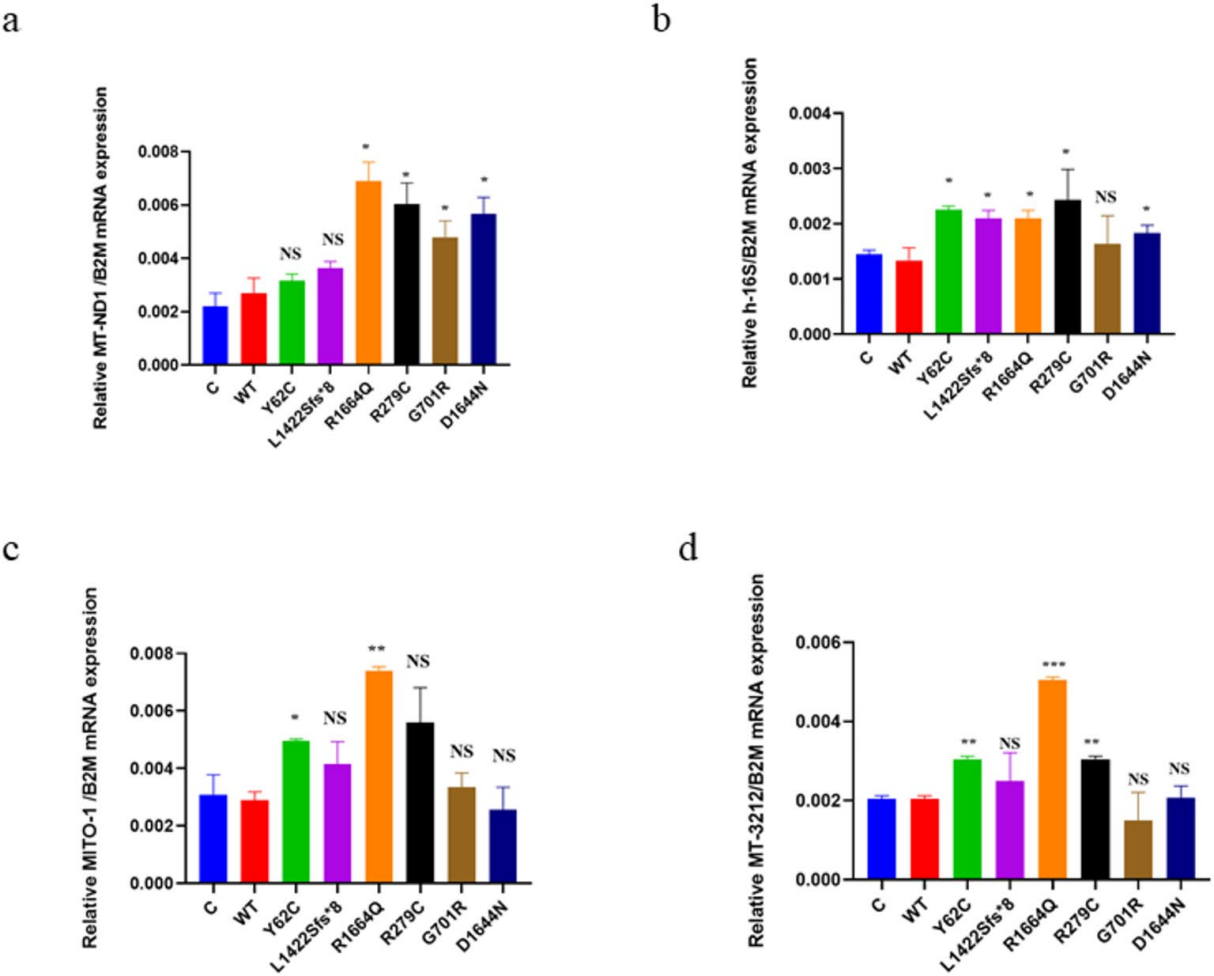
Mitochondrial copy numbers results in the HEK293 cells. A two-unpaired parametric t-test t was used for the comparison between WT and each mutant. **a** Histogram showing relative MT-ND1/B2M copy numbers expression. **b** Histogram showing relative h-16 s/B2M copy numbers expression. **c** Histogram showing relative hMITO-1/B2M copy numbers expression. **d** Histogram showing relative MT-3212/B2M copy numbers expression. Values represent mean ± SEM of 9 experiments each. * *P* < 0.05, ** *P* < 0.01, *** *P* < 0.001, NS, non-significant; C, negative control; and WT, wild type

We also examined mitochondrial copy numbers. The Shapiro–Wilk test confirmed normal distribution of 9 replicates of *MT-ND1*/*B2M*, *h-16S*/*B2M, hMITO*-*1*/*B2M*, and *MT-3212*/*B2M* mitochondrial copy numbers data. Consequently, WT was compared to each variant using a two-unpaired parametric t-test. We observed that *MT-ND1*/*B2M* copy numbers were significantly higher than WT for p.R1664Q, p.R279C, p.G701R, and p.D1644N, while p.Y62C and p.L1422Sfs*8 showed no significant change (Fig. 6a). The *h-16S*/*B2M* copy numbers were significantly higher than WT for p.Y62C, p.L1422Sfs*8, p.R1664Q, p.R279C, and p.D1644N, but there was no significant change for p.G701R (Fig. 6b). The *hMITO*-*1*/*B2M* copy numbers were significantly higher than WT for p.Y62C and p.R1664Q, but there were no change for p.L1422Sfs*8, p.R279C, p.G701R, and p.D1644N (Fig. 6c). The copy numbers of *MT-3212*/*B2M* were statistically higher than WT for p.Y62C, p.R1664Q, and p.R279C, while there was no significant change for p.L1422Sfs*8, p.G701R, and p.D1644N (Fig. 6d). Therefore, it appears that elevated mitochondrial copy numbers are a compensatory mechanism against mitochondrial dysfunction.

**Fig. 7:**
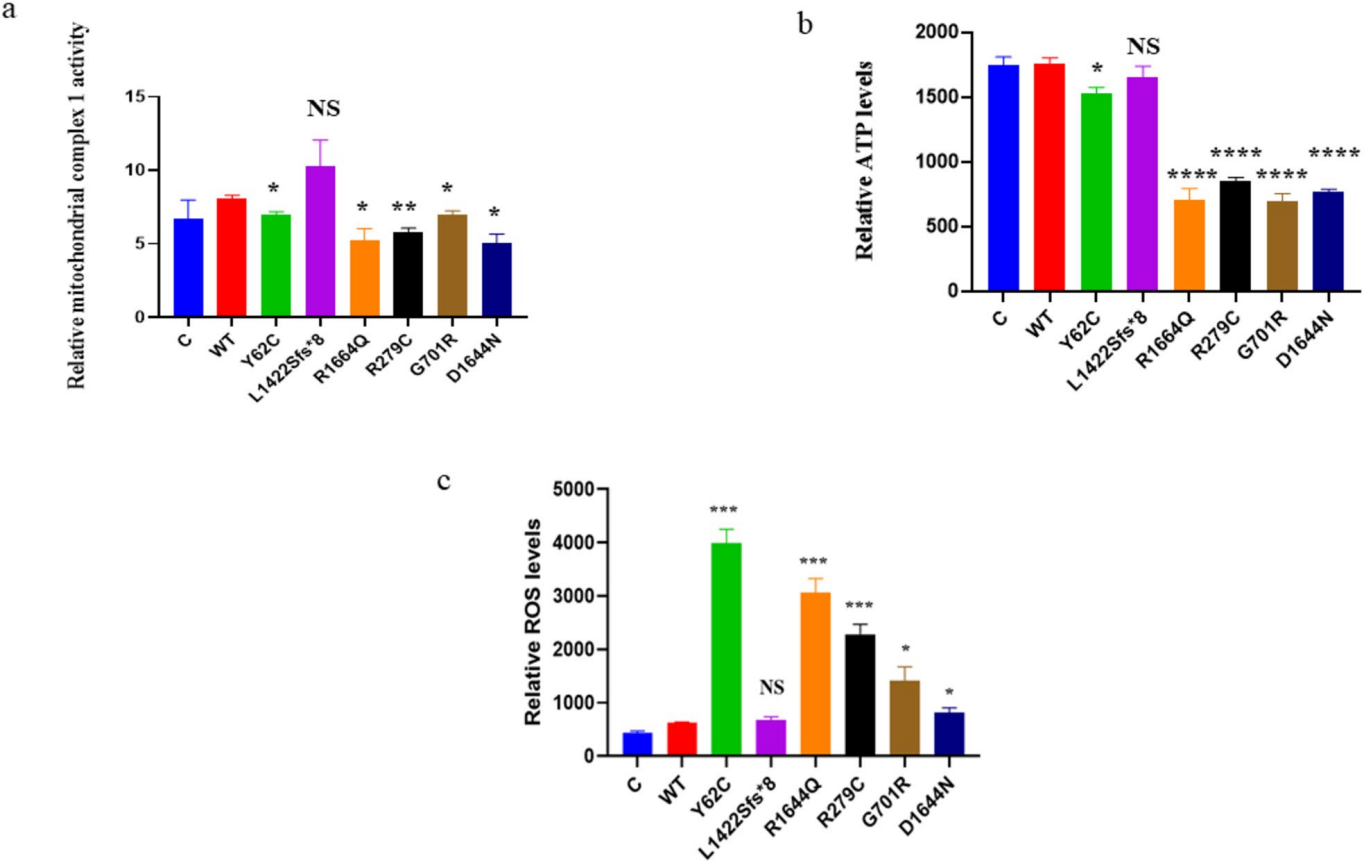
Mitochondrial complex I enzyme activity, ATP and ROS levels in the HEK293 cells. A two independent sample t-test was used for the comparison between WT and each mutant. **a** Histogram showing relative mitochondrial complex I enzyme activity. **b** Histogram showing relative ATP levels. **c** Histogram showing relative ROS levels. Values represent mean ± SEM of three experiments each. * *P* < 0.05, ** *P* < 0.01, *** *P* < 0.001, ****P < 0.0001, NS, non-significant; C, untransfected negative control; and WT, wild type

We also assessed mitochondrial complex 1 enzyme activity, ATP and ROS levels. WT was compared to each variant using a two-unpaired parametric t-test. Mitochondrial complex 1 enzyme activity was statistically significantly diminished for p.Y62C, p.R1664Q, p.R279C, p.G701R, and p.D1644N than WT, but there was no significant change for p.L1422Sfs*8 (Fig. 7a). The p.Y62C, p.R1664Q, p.R279C, p.G701R, and p.D1644N variants expressed lower ATP levels than WT, but there was no significant change for the p.L1422Sfs*8 variant (Fig. 7b). The p.Y62C, p.R1664Q, p.R279C, p.G701R, and p.D1644N variants exhibited significantly higher ROS levels than WT, but there was no significant change for the p.L1422Sfs*8 variant (Fig. 7c).

**Fig. 8:**
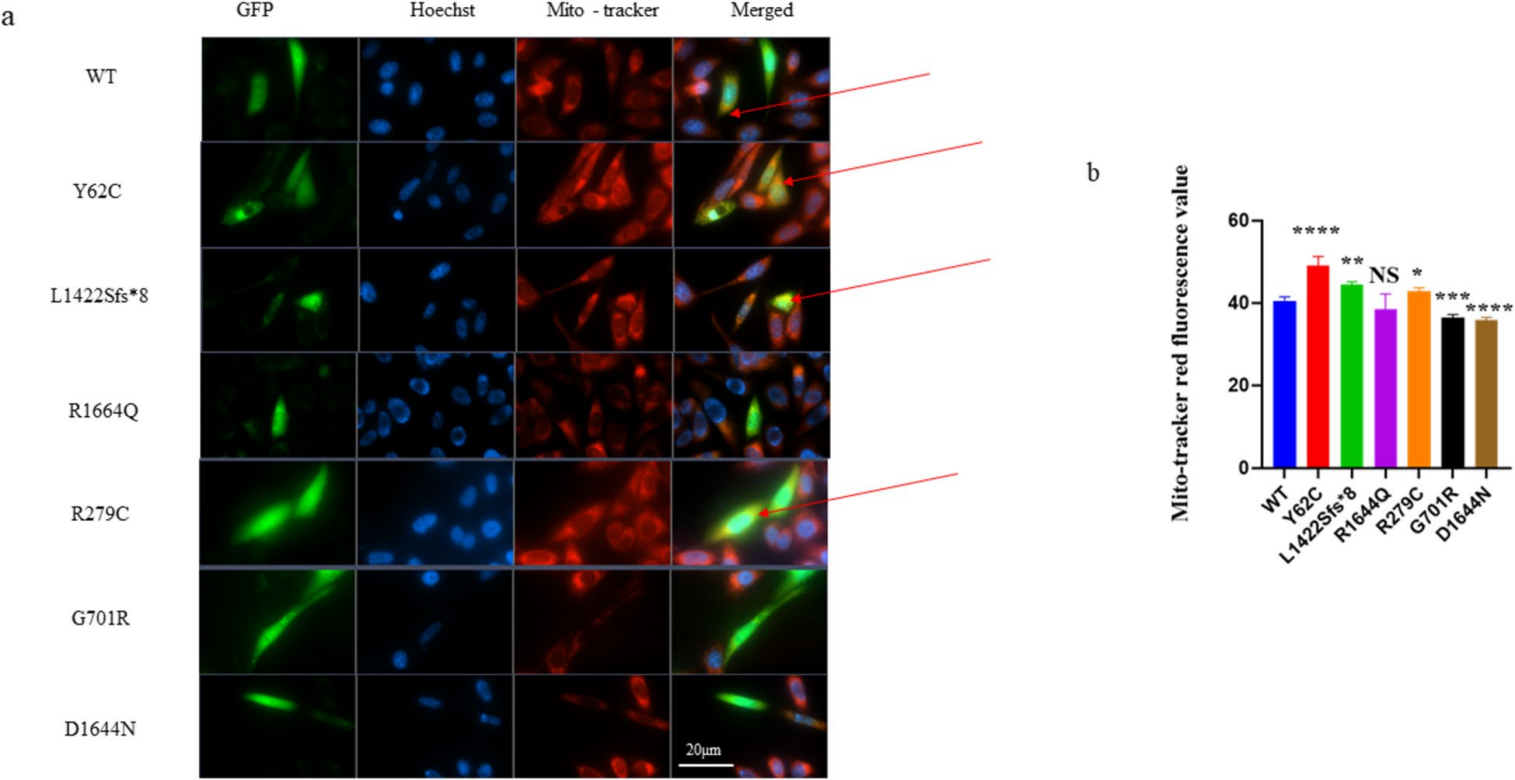
Fluorescence microscopy for the mito-tracker red staining in CHO cells. A two-unpaired parametric t-test was used for the comparison between WT and each mutant. **a** Ca_v_2.1 protein might co-localize with mitochondria in some variants as shown with red arrow. **b** Histogram showing relative mito-tracker red staining fluorescence.. Scale bar, 20 μm. NS, non-significant; WT, wild type. Data and images shown are representative of 4 independent experiments with at least 50 cells

We investigated the effects of variants on mitochondrial expression using CHO cells. After removing outliers from a sample of more than 50 cells per group, we found that the results were normally distributed according to the Anderson–Darling, D’Agostino and Pearson, Shapiro–Wilk, and Kolmogorov–Smirnov tests. Therefore, WT was compared to each variant using a two-sample parametric t-test. The Ca_v_2.1 protein might co-localize with the mitochondria (Fig. 8a). The p.Y62C, p.L1422Sfs*8, and p.R279C variants demonstrated statistically significant higher mitochondrial intensity than WT, while p.G701R and p.D1644N demonstrated statistically significant lower intensity than WT (Fig. 8b). However, there was no significant change for p.R1664Q (Fig. 8b).

**Fig. 9:**
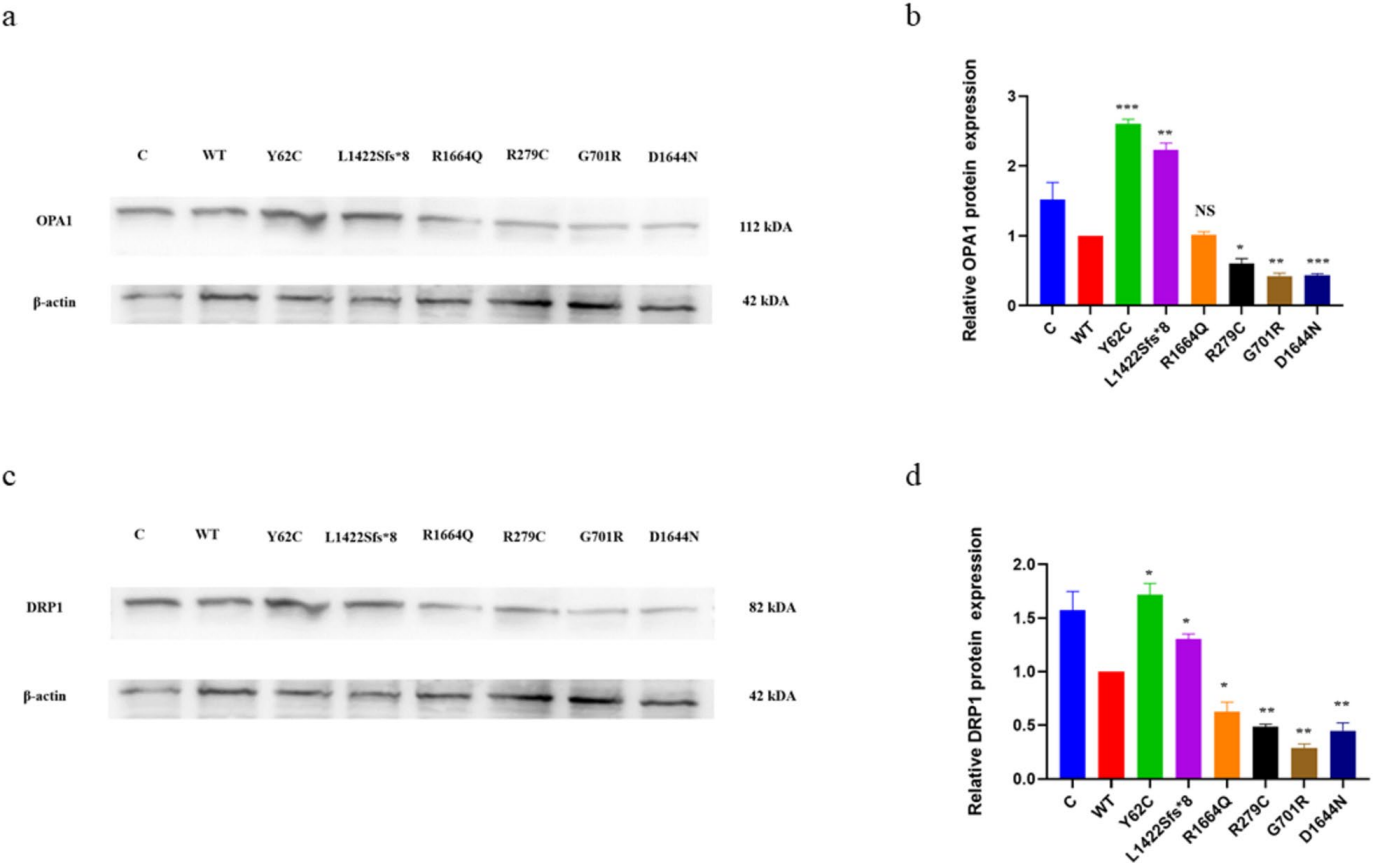
Western blot analysis for the mitochondrial fusion and fission markers in the HEK293 cells. A two-unpaired parametric t-test was used for the comparison between WT and each mutant. **a** Immublots of the relative OPA1 protein expression. **b** Histogram showing relative OPA1 protein expression. **c** Immublots of the relative DRP1 protein expression. **d** Histogram showing relative DRP1 protein expression. Values represent mean ± SEM of five experiments. * *P* < 0.05, ** *P* < 0.01, *** *P* < 0.001, NS, non-significant; C, untransfected negative control; and WT, wild type

The balance of mitochondrial fusion, facilitated by mitofusins and OPA1, and fission, mediated by DRP1, regulates mitochondrial morphology. The Shapiro–Wilk test confirmed normal distribution of five replicates of OPA1 and DRP1 protein expression data. Thereafter, WT was compared to each variant using a two-unpaired parametric t-test. The p.Y62C and p.L1422Sfs*8 variants showed significantly higher OPA1 protein expression than WT, while the p.R279C, p.G701R, and p.D1644N variants showed significantly lower OPA1 protein expression than WT (Fig. 9a, b) while the p.R1664Q showed no significant change (Fig. 9a, b). DRP1 protein expression was significantly higher for p.Y62C and p.L1422Sfs*8, but significantly lower for p.R1664Q, p.R279C, p.G701R, and p.D1644N (Fig. 9c, d).

**Fig. 10:**
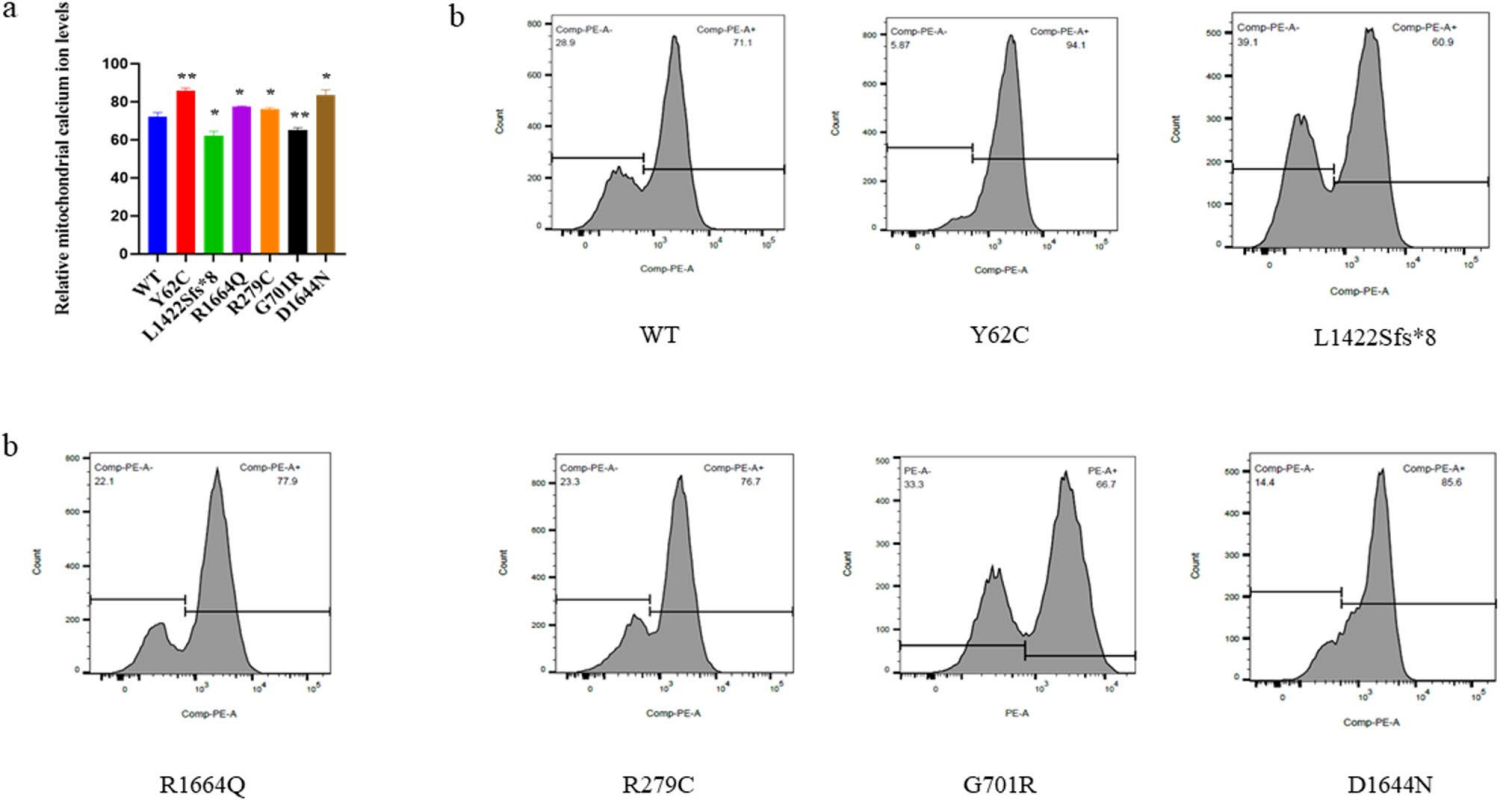
Mitochondrial calcium ions levels analysis by Rhod2-AM. A two-unpaired parametric t-test was used to compare mitochondrial calcium ion levels between WT and each mutant. **a** Histogram showing relative mitochondrial calcium ions levels. **b** Flow cytometry results for each variant. Values represent mean ± SEM of five independent experiments. * *P* < 0.05, ** *P* < 0.01, NS, non-significant; and WT, wild type

We also measured mitochondrial calcium ions levels. The Shapiro–Wilk test confirmed normal distribution of five replicates of mitochondrial calcium ion levels. Therefore, WT was compared to each variant using a two-unpaired parametric t-test. Consequently, we found that p.Y62C, p.R1664Q, p.R279C, and p. D1644N had statistically higher levels than WT, while p.L1422Sfs*8 and p.G701R had significantly lower levels (Fig. 10a, b).

### Variants impaired mitophagy and induced endoplasmic stress

**Fig. 11:**
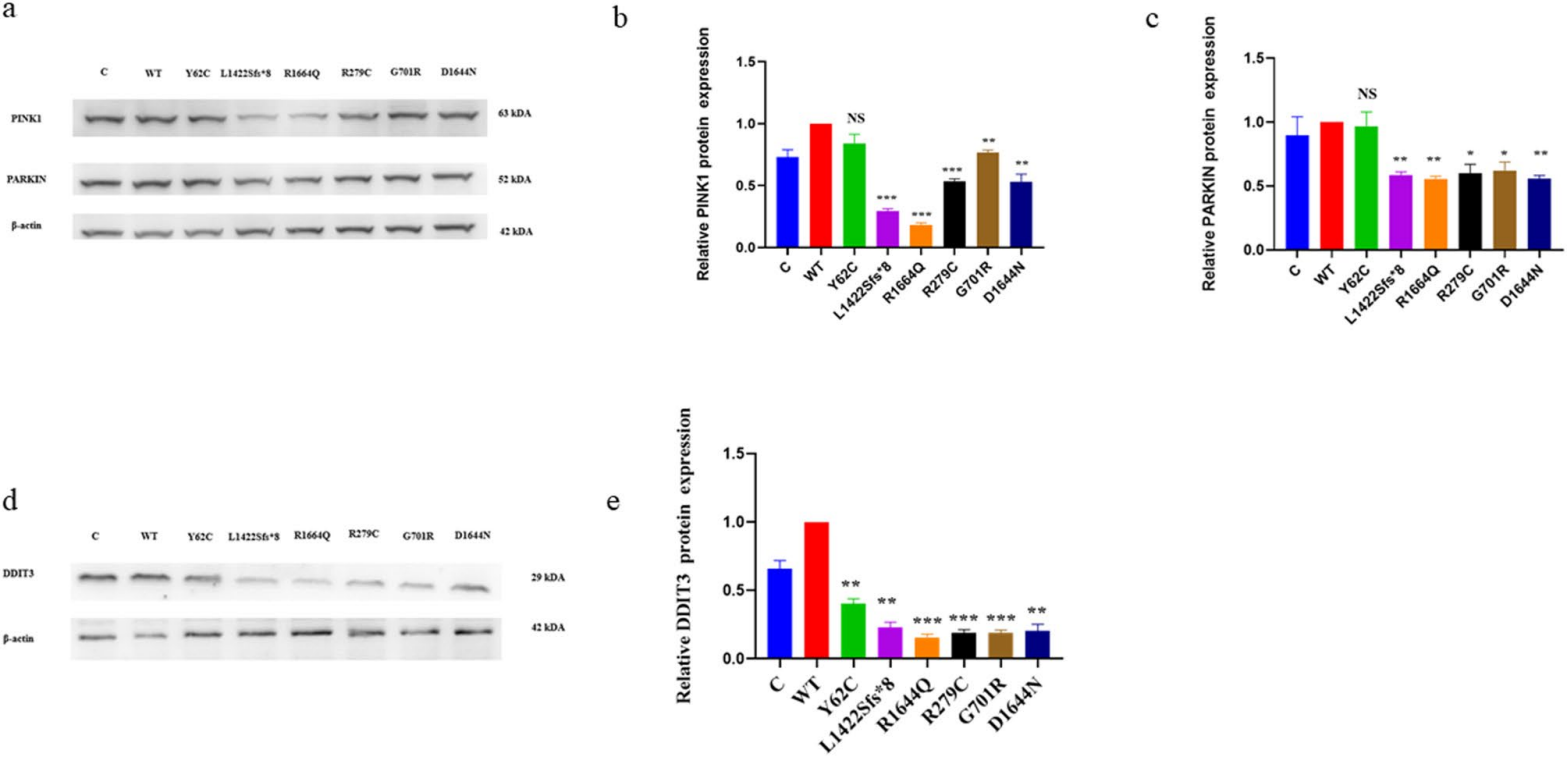
Western blot analysis for the mitophagy and endoplasmic reticulum stress markers in the HEK293 cells. A two-unpaired parametric t-test was used to compare between WT and each mutant. **a** Immublots of the PINK1 and PARKIN protein expression. **b** Histogram showing relative PINK1 protein expression. **c** Histogram showing relative PARKIN protein expression. **d** Immublots of the DDIT3 protein expression. **e** Histogram showing relative DDIT3 protein expression. Values represent mean ± SEM of five i experiments each. * *P* < 0.05, ** *P* < 0.01, *** *P* < 0.001, NS, non-significant; C, untransfected negative control; and WT, wild type

A reduction in mitophagy process accelerates the accumulation of dysfunctional mitochondria [43]. p62 inhibition decreases mitophagy, which is vital to the PINK1/PARKIN-mediated mitophagy pathway [43]. The Shapiro–Wilk test confirmed the normal distribution of the five replicate sets of data for PINK1, PARKIN, and DDIT3/CHOP protein expression. Thereafter, WT was compared to each variant using a two-unpaired parametric t-test. Consequently, the p.Y62C, p.L1422Sfs*8, p.R1664Q, p.R279C, and p.G701R variants expressed lower levels of p62 protein than WT. Besides, p.L1422Sfs*8, p.R1664Q, p.R279C, p.G701R, and p.D1644N had significantly lower PINK1 protein expression than WT, whereas p.Y62C showed no significant change (Fig. 11a, b). The p.L1422Sfs*8, p.R1664Q, p.R279C, p.G701R, and p.D1644N exhibited significantly lower PARKIN protein expression than WT, while p.Y62C showed no significant change (Fig. 11a–c). In summary, the p.L1422Sfs*8, p.R1664Q, p.R279C, p.G701R, and p.D1644N variants had an impaired mitophagy process. Additionally, all variants exhibited lower DDIT3/CHOP protein expression levels than WT (Fig. 11d, e), implying the presence of endoplasmic reticulum stress.

## Discussion

Calcium ions are involved in many physiological processes, which are vital for the cells, but they can also be cytotoxic for living organisms necessitating calcium-buffering system [51, 52]. Cytosolic calcium ions act as second messenger; an important mediator of signalling process between cells [51]. They activate many physiological actions including synaptic transmission, muscle contraction, hormone secretion and gene expression [53, 54]. Moreover, calcium ions regulate cell cycle from mitosis to apoptosis [55, 56]. Cytosolic calcium modulates neuronal firing pattern [57]. Dysregulation of calcium ions homeostasis and signalling in the cells and organelles play a crucial role in the pathogenesis of several diseases including cardiovascular diseases [58], Parkinson’s disease [59], bone diseases [60], immune system disease [61], lysosomal storage disease Niemann-Pick type C [62] as well as Alzheimer’s disease, Huntington’s disease, and epilepsy [63]. Mitochondria are also crucial for regulating ATP production, calcium regulation, ROS production, and apoptosis [64]. Notably, dysregulation of mitochondrial calcium handling in excitatory post-synaptic neurons can lead to Parkinson’s disease and amyotrophic lateral sclerosis [65].

This study examined the molecular mechanisms underlying the following *CACNA1A* variants: p.D1644N, p.Y62C, p.G701R, p.R279C, p.R1664Q, and p.L1422Sfs*8. Most variants affected mitochondria and lysosomes and induced endoplasmic reticulum stress. The p.Y62C variant’s phenotype is nearly identical to those reported in the previous studies [29, 30], which include focal epilepsy, status epilepticus, and GDD. The p.R279C variant has been reported in nine additional individuals with a wide range of phenotypes [34–36].^.^ The p.R1664Q variant has been reported in six additional cases with overlapping phenotypes [31, 32]. Our study corroborates previous reports, showing that cases harbouring *CACNA1A* variants typically present with ID/GDD, early-onset progressive ataxia, progressive cerebral atrophy, epilepsy, severe brain edema, coma, stroke-like presentations, and movement disorders. These manifestations are almost similar to those of mitochondrial diseases [6–10, 21, 22, 66–71].

Some studies have shown that electrophysiological studies alone cannot fully explain the complex clinical manifestations related to this gene. Although our study shows that the Ca_v_2.1 channel might be found on mitochondria, we could not investigate whether its function and regulation are independent of the Ca_v_2.1 channel located on the plasma membrane, as is the case with lysosomes [16]. It is also unclear whether cytoplasmic and mitochondrial calcium ion levels are the same; therefore, animal model studies are invited to investigate these unanswered facts. The mitochondrial complex I enzyme plays an important role in generating ATP. Thus, impairment of this enzyme can alter ATP production and lead to the production of ROS, resulting in mitochondrial damage and the generation of other complications, including the destruction of nucleic acids [18, 72, 73]. Our study has shown that mitochondrial complex I enzyme dysfunction can be found in *CACNA1A* variants in agreement with the previous reports of the two patients [21, 22]. In our study, all variants except one exhibited lower mitochondrial complex I enzyme activity and ATP levels than WT. The OPA1 protein is located in the inner mitochondrial membrane, where it regulates mitochondrial fusion and cristae morphology, as well as protecting against apoptosis. The DRP1 protein regulates mitochondrial functions by inhibiting mitophagy (suppressing the recruitment of Mito-Parkin) and enhancing apoptosis by promoting the mitochondrial translocation of Bax [74]. All of our variants expressed abnormal levels of OPA1 and DRP1 proteins, suggesting impaired mitochondrial fusion and fission. Besides, all variants expressed abnormal SDHA and MT-CO1 levels compared to WT, suggesting mitochondrial dysfunction. As a feedback mechanism that compensates for defects in mitochondria carrying mutated mtDNA, oxidative stress in human cells has been shown to upregulate mitochondrial copy numbers, as observed in our study [23, 75]. Similarly, mitochondrial dysfunction, including altered mitochondrial complex activities, impaired fusion and fission, and altered ATP and ROS levels, has been reported in *CACNA1C*-related disorders [28, 76, 77].

Mitophagy, a type of autophagy specific to mitochondria, is regulated by PINK1 and PARKIN. These proteins have protective functions in cells by regulating mitophagy and mitochondrial fission/fusion. They also prompt an elimination of impaired mitochondrial components and promote mitochondrial biogenesis and the translation of mitochondrial genes [78]. Studies have shown that PINK1/PARKIN deficiency leads to mitochondrial calcium overload and ROS production [78, 79], which is similar to our five variants. Although we could not explore much about effects of these variants in the endoplasmic reticulum, it worth noting that five of our variants expressed lower levels of the DDIT3/CHOP protein suggesting the presence of the endoplasmic reticulum stress [80]. Likewise, a recent study revealed that the α1A-polyQ and α1ACT-polyQ Ca_v_2.1 proteins can induce both the endoplasmic stress response and apoptosis implying that they can cause endoplasmic stress-induced apoptosis [81]. The α1ACT works via microRNA network to modulate neurogenesis and cell death during the development of the neonatal cerebellum [82]. Likewise, the compromised mitophagy process has been reported to play a role in some diseases such as Alzheimer’s diseases [83], Parkinson’s disease [84] and *CACNA1C*-related disorders [28].

Recently, it was revealed that Ca_v_2.1 channels are present on lysosomes, where they modulate fusion with autophagosomes and endosomes, which is crucial for neuronal homeostasis [16]. There is only one previous study that explored the effects of *CACNA1A* variants on lysosome; which revealed that variants disrupted lysosome calcium homeostasis in cerebellar neurons and caused endo-lysosomal fusion defect [17]. It has been shown that the regulation and functions of the Ca_v_2.1 channel found on the lysosomes is independent of the Ca_v_2.1 channel located on the plasma membrane [16]. Besides, it has been shown in the *Cacna1a* mice carrying spontaneous mutation that the cytoplasmic calcium levels are not the same as lysosomal calcium levels [17]. Consequently, this study provides more evidence that Ca_v_2.1 channel can be found on the lysosomes, and *CACNA1A* variants can lead to autophagy-lysosomal system dysfunctions: defective autophagy, blockage of the autophagosome-lysosome fusion, and disrupt lysosomal expression.

Notably, the p.L1422Sfs*8 variant’s phenotype was mainly EA2. This variant can lead to premature truncation. Amino acid position 1422 is in domain III S5. In a homozygous condition, like the HEK293 and CHO transfections, no functional Ca_v_2.1 would be made because the truncation does not allow the entire pore-forming region to exist. Therefore, this variant acts as a complete LOF control. *CACNA1A* encodes both the Ca_v_2.1 and α1ACT (a transcription factor). α1ACT is known to facilitate cerebellar neuronal development in both mice and humans[85]. It also facilitates neurogenesis and development of cerebellar dendritic synapses in the mice [82]. It has been shown that, under α1ACT modulation, up-/downregulated miRNA clusters with their paired target genes in mice may form a regulatory network controlling the balance between the neuronal proliferation, differentiation, and cell death in the cerebellum to promote neonatal development [82]. Since p.L1422Sfs*8 is so early that we could also lose the α1ACT transcription factor, we speculate that impaired cerebellar neuronal development and apoptosis might drive the phenotype in this mutant (EA2). The p.Y62C variant is located in the cytoplasmic linker of domain I S1, p. D1644N in the domain IV S4 transmembrane helix, p. G701R variant in the domain II S6 transmembrane helix, p. R279C variant in the domain I extracellular region of S5, and p. R1664Q in the domain IV S4 transmembrane helix. The movement of calcium ions in and out of the cell is regulated by S4, and the loop between S5 and S6 form the selectivity filter that controls calcium ions influx and efflux. Therefore, in addition to our findings, we speculate that the phenotypes of the p. D1644N, p. G701R, p. R279C, and p.R1664Q might have resulted from impaired calcium hemostasis in neurons leading to either neuronal hyperexcitability or hypo excitability. Although we could not explore these mechanisms in this study, we are looking forward to confirm them in mice in a near future.

It is worth noting that impairment of oxidative phosphorylation (OXPHOS) and cytochrome c oxidase (COX, complex IV)-negative fibers can drive mitochondrial deficits in muscle resulting to mitochondrial myopathies [86]. Mitochondrial deficits can involve several body muscles including skeletal, cardiac and smooth muscles. They can involve many body systems or organs including nervous system (patients can present with seizures, tremor, GDD/ID, deafness, stroke-like episodes, ataxia and peripheral neuropathy), skeletal muscles (patients can present with muscle weakness, exercise intolerance and cramps), eyes (patients can present with ptosis, external ophthalmoplegia and optic atrophy) and heart (patients can present with cardiomyopathy)[86]. Other additional involved organs or systems include, kidneys (patients can present with Fanconi syndrome, kidney failure and nephrotic syndrome), pancreas (patients can present diabetes), liver (patients can present with liver failure, and hepatic steatosis), and gastrointestinal (patients can present with gut dysmotility, intestinal pseudo-obstruction and chronic diarrhea)[86]. However, skeletal muscle are commonly affected and used for biopsy purposes [86]. We speculate that there could overlapping mechanisms at play in muscles and neurons where the calcium channel (Ca_v_2.1) is expressed and mitochondria is in a pathological state. Some of the overlapping mechanism could be impairment of OXPHOS. Nevertheless, mitochondrial deficits in neurons can have more complex mechanisms, which we could not investigate in this study including impairment of neuronal excitability and transmission resulting to complex neurological diseases like epilepsy, Parkinson’s disease, Alzheimer’s disease, Huntington’s disease and amyotrophic lateral sclerosis as summarized above.

## Conclusion

*CACNA1A* variants may alter mitochondrial and lysosomal functions. This process may be involved in the pathogenesis of *CACNA1A*-related NDDs.

### Limitations

We were unable to conduct several experiments, including checking other mitochondrial complex activities and mitochondrial potential. We were unable to perform transmission electron microscopy to visualize mitochondrial morphology. We used HEK293 and CHO cells as models, but they do not accurately represent the *CACNA1A* phenotype physiologically. Additionally, HEK293 cells are highly aneuploid. We used a transient transfection protocol, which can lead to excessive protein production. Additionally, using t2A-EGFP2 plasmids with a self-cleaving site (t2A) may have altered mito-tracker and lyso-tracker results. The effects of human variants may not be reflected by simply expressing a mutant construct. Although our study is the first study to show that the Ca_v_2.1 channel can be found on mitochondria, it could not investigate whether its function and regulation are independent of the Ca_v_2.1 channel located on the plasma membrane. Although endoplasmic reticulum is rather the most widely storage of calcium ions, we did not do many experiments on it. Our study was conducted only in cell models, so animal model studies involving neurons are needed to verify these findings. In the near future, we will consider using patient-derived cell models, including isogenic WT-mutant pairs generated by CRISPR/Cas9 editing and perform more experiments including those related to endoplasmic reticulum.

## Supplementary Information

Supplementary file 1.

Supplementary file 2.

Supplementary file 3.

Supplementary file 4.

Supplementary file 5.

Supplementary file 6.

## Data Availability

The datasets generated and/or analyzed during the current study are available in the article/Supplementary Materials, further inquiries can be directed to the corresponding author.
