# Supplementary material for: Mitochondrial and lysosomal dysfunctions might be involved in the pathogenesis of the CACNA1A-related neurodevelopmental disorders according to in vitro studies
Source: Biol Res. 2025 Dec 27;58:76. doi: 10.1186/s40659-025-00655-w (PMC12751537; doi:10.1186/s40659-025-00655-w)
Supplement: Supplementary file 1 [file 40659_2025_655_MOESM1_ESM.pdf]

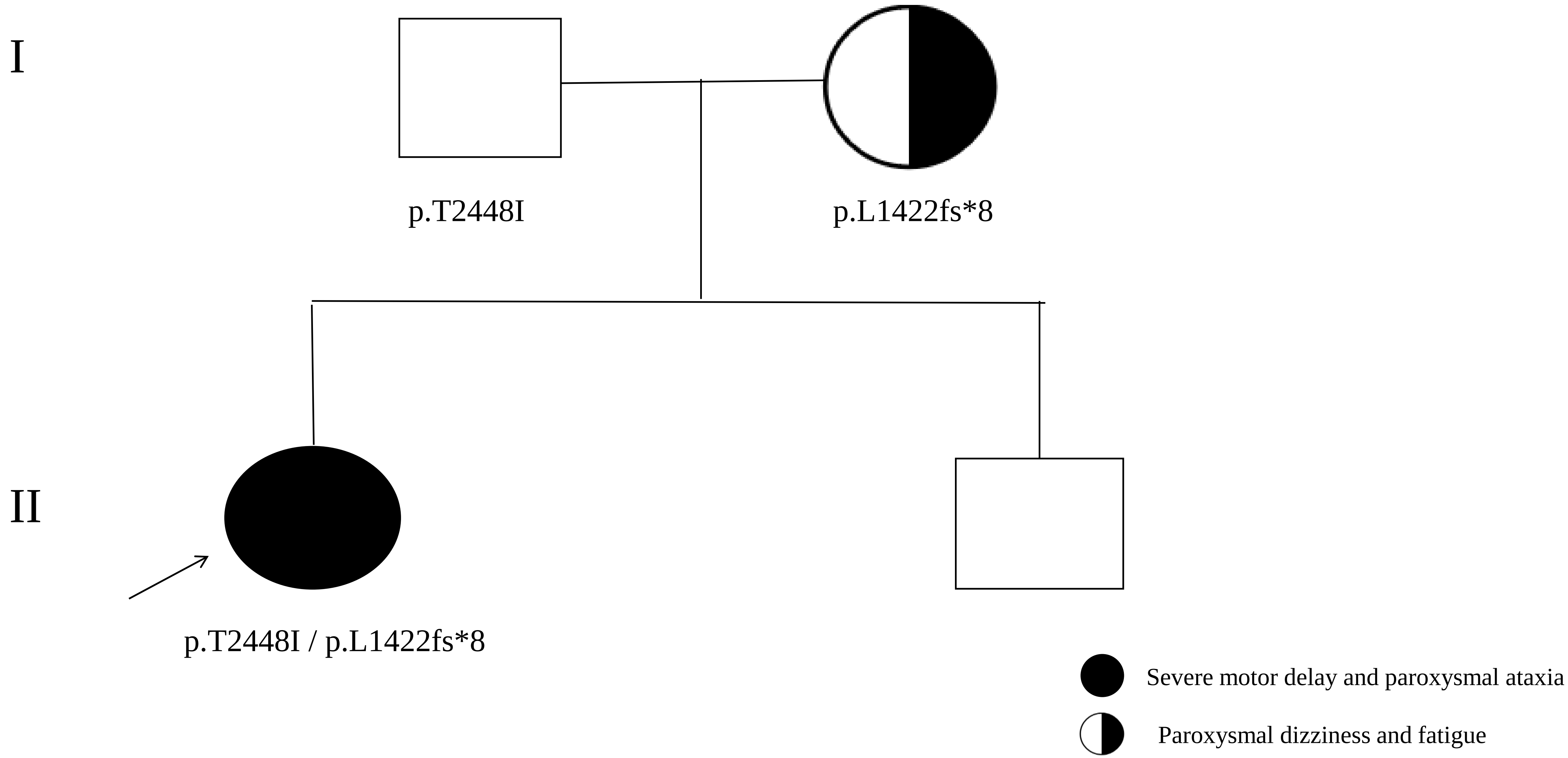
I
p.T2448I
p.L1422fs*8
II
p.T2448I / p.L1422fs*8
Severe motor delay and paroxysmal ataxia
Paroxysmal dizziness and fatigue
