# Supplementary material for: Mitochondrial and lysosomal dysfunctions might be involved in the pathogenesis of the CACNA1A-related neurodevelopmental disorders according to in vitro studies
Source: Biol Res. 2025 Dec 27;58:76. doi: 10.1186/s40659-025-00655-w (PMC12751537; doi:10.1186/s40659-025-00655-w)
Supplement: Supplementary file 2 [file 40659_2025_655_MOESM2_ESM.docx]

**Supplementary Table 1:** List of the primers used in this study.

| **Name of the primer** | **Primer sequence (5’- 3’)** |
| --- | --- |
| Human beta-actin-F | 5’ AGAGCTACGAGCTGCCTGAC3’ |
| Human beta-actin-R | 5’ AGCACTGTGTTGGCGTACAG 3’ |
| Human CACNA1A-F | 5’TATGAAAATGCCCTGCGGGT3’ |
| Human CACNA1A-R | 5’TCTCGAGTCAGGATGCCAGA3’ |
