# Supplementary material for: Mitochondrial and lysosomal dysfunctions might be involved in the pathogenesis of the CACNA1A-related neurodevelopmental disorders according to in vitro studies
Source: Biol Res. 2025 Dec 27;58:76. doi: 10.1186/s40659-025-00655-w (PMC12751537; doi:10.1186/s40659-025-00655-w)
Supplement: Supplementary file 3 [file 40659_2025_655_MOESM3_ESM.docx]

**Supplementary Table 2**: List of the antibodies used in this study.

| Name | Specifications | Dilution | Brand name |
| --- | --- | --- | --- |
| Anti-CACNA1A mouse antibody | ab181371 | 1:5000 | Abcam |
| Anti-beta Actin mouse antibody | ab8226 | 1:10000 | Abcam |
| Anti -GAPDH Rabbit pAb | GB11002 | 1:1000 | Servicebio |
| HRP Conjugated AffiniPure Goat Anti-mouse IgG (H+L) | BA1050 | 1:5000 | BOSTER |
| HRP Conjugated AffiniPure Goat Anti-rabbit IgG (H+L) | BA1054 | 1:5000 | BOSTER |
| Beclin-1 rabbit antibody | 3738 | 1:1000 | Cell signaling Technology |
| SQSTM1/P62 rabbit antibody | R25788 | 1:1000 | ZENBIO |
| Anti‑LC3B rabbit antibody | E7X4S | 1:1000 | Cell signaling Technology |
| LAMP-1 anti-mouse antibody (H4A3) | sc-20011 | 1:100 | SANTA CRUZ BIOTECHNOLOGY, INC |
| PINK1 anti-rabbit antibody | 507131 | 1:1000 | ZENBIO |
| Parkin rabbit antibody | 381626 | 1:1000 | ZENBIO |
| Anti-OPA1 rabbit antibody (JM81-35) | ET1705-9 | 1:1000 | Hangzhou HuaAn Biotechnology |
| DDIT3 rabbit antibody | R23316 | 1:1000 | ZENBIO |
| DRP1 mouse antibody | 221099 | 1:1000 | ZENBIO |
| Anti-MTCO1 mouse antibody [1D6E1A8] | ab14705 | 1:1000 | Abcam |
