# Supplementary material for: Mitochondrial and lysosomal dysfunctions might be involved in the pathogenesis of the CACNA1A-related neurodevelopmental disorders according to in vitro studies
Source: Biol Res. 2025 Dec 27;58:76. doi: 10.1186/s40659-025-00655-w (PMC12751537; doi:10.1186/s40659-025-00655-w)
Supplement: Supplementary file 5 [file 40659_2025_655_MOESM5_ESM.docx]

**Supplementary Table 4**

**Clinical characteristics of 6 patients with *CACNA1A*-related neurodevelopmental disorders in this study**

| **Patients** | **P1** | **P2** | **P3** | **P4** | **P5** | **P6** |
| --- | --- | --- | --- | --- | --- | --- |
| **Mutation** | c.185A>G, p.Y62C | c.4264delC, p. L1422Sfs*8 | c.4991G>A, p. R1664Q | c.835C>T, p. R279C | c.2101G>A, p. G701R | c.4930G>A, p. D1644N |
| **Age/Sex** | 3y2mo/M | 9y10mo/F | 8y8mo/F | 9y8mo/F | 5y11mo/M | 13y10mo/M |
| **Age of seizure onset** | 1y20d | - | - | 5y11mo | - | 10y |
| **Seizure semiology** | Focal seizures | - | - | Absence seizures | - | Focal seizures |
| **Seizure frequency** | Once in 2 weeks | - | - | 1-2 times per day | - | More than 10 days |
| **Age of seizure control** | - | - | - | Ongoing seizures, 1-2 times per day | - | Seizure attack after 1-2 weeks |
| **Presence of the status epilepticus** | Yes | - | - | No | - | No |
| **History of febrile seizures** | Yes. Seizures occurred mainly in high fever, (38.5-39 °C) and was accompanied by chills | - | - | Yes. 3 attacks with temperature >39℃ | - | No |
| **EEG findings** | During sleep, the sharp and slow waves in the frontal, central and temporal regions were distributed on the left hemisphere. | Bilateral slow waves and slow waves burst on the occipital area | Normal | During sleep, 3-4 Hz spike slow waves and multi-spike slow waves. Delta slow waves emission in frontal, occipital and midline regions during wakefulness | 3-4 Hz slow waves, paroxysm / rhythmic emission in temporal region | Generalized or multifocal spike and slow waves |
| **Brain MRI** | Abnormal signal in the right hippocampus. The left hippocampus was slightly flat and the temporal horn of bilateral ventricles were enlarged | Right choroidal fissure cyst | Progressive cerebellar atrophy | Normal | Cerebellar atrophy and large left ventricular temporal horn | Cerebellar atrophy, mild enlargement of right ventricle, temporal horn and occipital cistern. |
| **Patients** | **P1** | **P2** | **P3** | **P4** | **P5** | **P6** |
| **Treatment strategies** | Sodium valproate and carbamazepine | - | Acetazolamide | Lamotrigine, sodium valproate and acetazolamide | - | Oxcarbazepine, levetiracetam, and ketogenic diet |
| **Is there a developmental delay before the onset of epilepsy?** | Yes | No | Yes | No | Yes | Yes |
| **Severity of ID/GDD** | Severe | Normal | Mild | Mild | Profound | Profound |
| **Motor development** | Has unstable walk (progressive ataxia) | EA2 | Can walk alone but easy to fall and cannot run. Progressive ataxia. | Poor coordination of independent walking posture (progressive ataxia). | Can neither seat nor walk alone. EA2 | Abnormal gait, dragging on the left side (stroke like), cannot run, jump and severe motor delay. Progressive ataxia. |
| **Language development** | Unconsciously can call mom and dad | Normal | Repetitive words | Can speak slowly and clear | Can simply express their ideas but inarticulate | Can communicate easily, can say sentences, recite poems, but inarticulate |
| **Family history** | There is a family history of epilepsy | Mother has mild phenotype; paroxysmal dizziness and fatigue | No | No | No | There is a family history of epilepsy |
| **Other clinical features** | ASD. Unstable walking and loss of consciousness and progressive ataxia | Severe motor delay, paroxysmal walking difficulties and EA2 | Progressive ataxia | Dizziness, loss of consciousness and memory, paroxysmal limb weakness, and walk instability (EA2) | ASD, progressive ataxia | Fainting attacks and loss of consciousness. Progressive ataxia |
| **Physical examination** | Large area of coffee spot on the right arm, incomplete descending of the left testicle, normal muscle strength and muscle tone of the limbs | Normal muscle strength and tone of limbs | Nystagmus, wide base gait, uncoordinated, easy to fall, normal muscle strength, slightly lower muscle tone, and reduced bilateral knee reflexes | Normal muscle tone of limbs, difficulty in closing eyes, slightly unsteady on finger and nose | There was a 2 * 3cm coffee spot on the left abdomen, slight nystagmus, pectus excavatum and hypotonia bilatearally | Left side, grade IV, hypotonia |

**Abbreviations**: D: day, EEG: electroencephalograph, EA2: episodic ataxia 2, F: female, GDD: global developmental delay, I: intellectual disability, M: male, Mo: month, MRI: magnetic resonance imaging, Y: year,
