# Supplementary material for: Mitochondrial and lysosomal dysfunctions might be involved in the pathogenesis of the CACNA1A-related neurodevelopmental disorders according to in vitro studies
Source: Biol Res. 2025 Dec 27;58:76. doi: 10.1186/s40659-025-00655-w (PMC12751537; doi:10.1186/s40659-025-00655-w)
Supplement: Supplementary file 6 [file 40659_2025_655_MOESM6_ESM.docx]

**Supplementary Table 5**

**Genetic results summary of 6 patients with *CACNA1A*-associated neurodevelopmental disorders in this study**

|  | **P1** | **P2** | **P3** | **P4** | **P5** | **P6** |
| --- | --- | --- | --- | --- | --- | --- |
| **Chromosome position (Hg 19)** | chr19:13616854 | chr19:13370505 | Chr19:13235693 | chr19:13470563 | chr19:13414587 | chr19:13356019 |
| **Nucleic acid alteration** | c.185A>G | c.4264delC | c.4991G>A | c.835C>T | c.2101G>A | c.4930G>A |
| **Amino acid change** | p. Y62C | p. L1422Sfs*8 | p. R1664Q | p. R279C | p. G701R | p. D1644N |
| **Transcript** | NM_001127221 | NM_001127221.1 | NM_001127221.2 | NM_023035 | NM_023035 | NM_001127221.1 |
| **Exon / intron** | exon1 | exon27 | exon32 | exon6 | exon16 | exon 31 |
| **Structural domain** | I | III | IV | I | II | IV |
| **Heterozygosity** | Heterozygous | Heterozygous | Heterozygous | Heterozygous | Heterozygous | Heterozygous |
| **Genetic pattern** | AD | AD | AD | AD | AD | AD |
| **Mode of inheritance** | *De novo* | Inherited from the mother with mild symptoms | *De novo* | Inherited from the unaffected father | *De novo* | *De novo* |
| gnomADV3 | Not recorded | Not recorded | Not recorded | Not recorded | Not recorded | Not recorded |
| gnomADV2.11 | Not recorded | Not recorded | Not recorded | Not recorded | Not recorded | Not recorded |
| Previous publications | (Zhang et al., 2020, Niu et al., 2022) | No | (Martínez-Monseny et al., 2021, Luo et al., 2017) | (Le Roux et al., 2021, Angelini et al., 2019, Lipman et al., 2022) | No | No |
| **Mutation taster** | Disease causing | Disease causing | Disease causing | Disease causing | Disease causing | Disease causing |
| **POLYPHEN** | Probably damaging  (score：0.999) | - | Probably damaging  (score：1.000) | Possibly damaging  (score：0.939) | Probably damaging  (score：1.000) | Probably damaging  (score：1.000) |
| **PROVEAN**  **PROTEIN** | Deleterious  (score：- 6.542) | - | - | Deleterious  (score：-5.780) | - | - |
| **Previous functional study results (reference)** | Predicted as GOF (Zhang et al., 2020) | None | LOF (Luo et al., 2017) | LOF (Le Roux et al., 2021) | None | None |
| **ACMG score** | LP | VUS | P | P | LP | LP |
| **Current functional study results** | GOF | LOF | LOF | LOF | LOF | GOF |
| **Final classification** | P | P | P | P | P | P |

Abbreviations: AD: autosomal dominant, GOF: gain-of- function, LOF: loss-of-function, P: pathogenic, LP: likely pathogenic, VUS: variant of unknown significance.

**References**

ANGELINI, C., VAN GILS, J., BIGOURDAN, A., JOUK, P. S., LACOMBE, D., MENEGON, P., MOUTTON, S., RIANT, F., SOLE, G., TOURNIER-LASSERVE, E., TRIMOUILLE, A., VINCENT, M. & GOIZET, C. 2019. Major intra-familial phenotypic heterogeneity and incomplete penetrance due to a CACNA1A pathogenic variant. *Eur J Med Genet,* 62**,** 103530.

LE ROUX, M., BARTH, M., GUEDEN, S., DESBORDES DE CEPOY, P., AEBY, A., VILAIN, C., HIRSCH, E., DE SAINT MARTIN, A., PORTES, V. D., LESCA, G., RIQUET, A., CHATON, L., VILLENEUVE, N., VILLARD, L., CANCES, C., VALTON, L., RENALDO, F., VERMERSCH, A. I., ALTUZARRA, C., NGUYEN-MOREL, M. A., VAN GILS, J., ANGELINI, C., BIRABEN, A., ARNAUD, L., RIANT, F. & VAN BOGAERT, P. 2021. CACNA1A-associated epilepsy: Electroclinical findings and treatment response on seizures in 18 patients. *Eur J Paediatr Neurol,* 33**,** 75-85.

LIPMAN, A. R., FAN, X., SHEN, Y. & CHUNG, W. K. 2022. Clinical and genetic characterization of CACNA1A-related disease. *Clinical genetics,* 102**,** 288-295.

LUO, X., ROSENFELD, J. A., YAMAMOTO, S., HAREL, T., ZUO, Z., HALL, M., WIERENGA, K. J., PASTORE, M. T., BARTHOLOMEW, D., DELGADO, M. R., ROTENBERG, J., LEWIS, R. A., EMRICK, L., BACINO, C. A., ELDOMERY, M. K., COBAN AKDEMIR, Z., XIA, F., YANG, Y., LALANI, S. R., LOTZE, T., LUPSKI, J. R., LEE, B., BELLEN, H. J. & WANGLER, M. F. 2017. Clinically severe CACNA1A alleles affect synaptic function and neurodegeneration differentially. *PLoS Genet,* 13**,** e1006905.

MARTÍNEZ-MONSENY, A. F., EDO, A., CASAS-ALBA, D., IZQUIERDO-SERRA, M., BOLASELL, M., CONEJO, D., MARTORELL, L., MUCHART, J., CARRERA, L., ORTEZ, C. I., NASCIMENTO, A., OLIVA, B., FERNÁNDEZ-FERNÁNDEZ, J. M. & SERRANO, M. 2021. CACNA1A Mutations Causing Early Onset Ataxia: Profiling Clinical, Dysmorphic and Structural-Functional Findings. *Int J Mol Sci,* 22.

NIU, X., YANG, Y., CHEN, Y., CHENG, M., LIU, M., DING, C., TIAN, X., YANG, Z., JIANG, Y. & ZHANG, Y. 2022. Genotype-phenotype correlation of CACNA1A variants in children with epilepsy. *Developmental medicine and child neurology,* 64**,** 105-111.

ZHANG, L., WEN, Y., ZHANG, Q., CHEN, Y., WANG, J., SHI, K., DU, L. & BAO, X. 2020. CACNA1A Gene Variants in Eight Chinese Patients With a Wide Range of Phenotypes. *Front Pediatr,* 8**,** 577544.
